# Dynein activating adaptor BICD2 controls radial migration of upper-layer cortical neurons in vivo

**DOI:** 10.1186/s40478-019-0827-y

**Published:** 2019-10-26

**Authors:** Lena Will, Sybren Portegies, Jasper van Schelt, Merel van Luyk, Dick Jaarsma, Casper C. Hoogenraad

**Affiliations:** 1 Department of Biology, Faculty of Science, Cell Biology, Neurobiology and Biophysics, Utrecht University, Padualaan 8, 3584 CH Utrecht, The Netherlands; 2 Department of Neuroscience, Erasmus MC, Rotterdam, 3015 GD The Netherlands; 3 Department of Neuroscience, Genentech, Inc, South San Francisco, CA 94080 USA

**Keywords:** BICD2, Radial neuronal migration, Neocortical development, Dynein adaptor

## Abstract

For the proper organization of the six-layered mammalian neocortex it is required that neurons migrate radially from their place of birth towards their designated destination. The molecular machinery underlying this neuronal migration is still poorly understood. The dynein-adaptor protein BICD2 is associated with a spectrum of human neurological diseases, including malformations of cortical development. Previous studies have shown that knockdown of BICD2 interferes with interkinetic nuclear migration in radial glial progenitor cells, and that *Bicd2*-deficient mice display an altered laminar organization of the cerebellum and the neocortex. However, the precise in vivo role of BICD2 in neocortical development remains unclear. By comparing cell-type specific conditional *Bicd2* knock-out mice, we found that radial migration in the cortex predominantly depends on BICD2 function in post-mitotic neurons. Neuron-specific *Bicd2* cKO mice showed severely impaired radial migration of late-born upper-layer neurons. BICD2 depletion in cortical neurons interfered with proper Golgi organization, and neuronal maturation and survival of cortical plate neurons. Single-neuron labeling revealed a specific role of BICD2 in bipolar locomotion. Rescue experiments with wildtype and disease-related mutant BICD2 constructs revealed that a point-mutation in the RAB6/RANBP2-binding-domain, associated with cortical malformation in patients, fails to restore proper cortical neuron migration. Together, these findings demonstrate a novel, cell-intrinsic role of BICD2 in cortical neuron migration in vivo and provide new insights into BICD2-dependent dynein-mediated functions during cortical development.

## Highlights

- Neuron-specific conditional *Bicd2* knockout mice show severe cortical neuronal migration defects
- Cell-intrinsic function of BICD2 is essential for nuclear migration during locomotion of upper-layer neurons, neuronal maturation and survival
- Mutant BICD2, associated with cortical malformation in patients, fails to rescue neuron-specific migration defects
- Glia-specific loss of BICD2 affects tempo-spatial regulation of RGP mitosis

## Introduction

A major challenge in neocortical development is to recruit diverse cell types into their proper layers and circuitries [27]. This is illustrated by the fact that multiple cortical malformation disorders exhibit an altered laminar organization of the cortex [17, 45, 54]. Neocortical development can roughly be divided into two major steps. First, diverse neocortical neurons are generated from progenitor cells within the ventricular and subventricular zones (VZ and SVZ). Radial glial progenitors (RGPs) first undergo self-renewal, before progressively switching to asymmetric division, producing one daughter RGP, and one daughter cell which is determined to become a neuron [40]. Mitosis only occurs if the RGP nucleus has migrated down to the apical ventricular surface in a movement known as interkinetic nuclear migration (INM) [21]. After asymmetric cell division, one of the daughter cells detaches from the ventricular surface and migrates to the SVZ. There, most become intermediate basal progenitors (iBPs) before dividing symmetrically to generate cortical projection neurons.

The second step in neocortical development is the movement of cells from their place of birth to their final destination. This process can be described as a sequence of three modes of migration, correlated with different cellular morphology of the nascent neurons [28, 42]. First, the newborn neurons acquire a multipolar morphology and migrate in random directions in the VZ and SVZ [38, 57], before moving towards the subplate (SP). In the upper intermediate zone (IZ), they gradually convert into bipolar cells by forming one long trailing process, which later becomes the axon. Additionally, a single leading edge is extended in the direction of the pia, giving rise to the future dominant dendrite. Following this transition, the bipolar neurons enter the CP and migrate in a locomotion mode towards the pia by using the basal processes of RGPs as a guide for radial migration [26, 42]. During bipolar locomotion, the leading edge of the neuron grows continuously towards the pial surface, while the nucleus follows in a saltatory fashion [59]. It has been proposed that translocation of the centrosome and subsequent nuclear movement via cytoskeleton remodeling and motor protein activity is essential for radial bipolar migration in the CP [14, 37, 59]. Finally, neurons complete their radial migration and execute glia-independent terminal somal translocation and initiate maturation. In the last two decades, an increasing number of proteins have been found to play an essential role in these processes. One of these proteins is the dynein activating adaptor protein Bicaudal-D2 (BICD2). So far, studies have shown that BICD2 is involved in RGP-related processes such as INM. However, the role of BICD2 in the migration of post-mitotic cortical neurons remains largely unclear.

Bicaudal-D2 (BICD2) is a dynein activating adaptor protein that plays a critical role in microtubule-based minus-end-directed transport. Motor adaptors allow for cargo-specific regulation of the dynein motor complex [44]. BICD2 activates dynein by enhancing the stability of the complex with dynactin, which leads to processive motility toward the microtubule minus end [19, 49]. In *Drosophila*, BicD was found to control nuclear positioning, endocytosis and lipid droplet transport, as well as dynein-mediated microtubule-dependent transport processes [6–8, 56]. Mammals possess two BicD orthologues: BICD1 and BICD2. Both these proteins are built from several coiled coil domains, which adopt a rod-like structure [55, 61]. The two N-terminal coiled coil domains of BICD2 bind to cytoplasmic dynein and dynactin [20], which has been shown to be important for activating the dynein motor complex. With its third C-terminal coiled coil domain (CC3), BICD2 binds to cargoes such as the small GTPase RAB6 and nucleoporin RANBP2. RAB6 localizes to the Golgi apparatus and exocytotic/secretory vesicles, and through these interactions BICD2 can contribute to Golgi organization and vesicle transport [16, 51]. In a cell-cycle regulated manner, BICD2 can switch from RAB6 to RANBP2 binding, which leads to dynein-dynactin recruitment to the nuclear envelope [52].

Mutations in the human BICD2 have been linked to a spectrum of neuronal disorders, in particular to a dominant mild early onset form of spinal muscular atrophy (SMALED2A: OMIM#615290) [35, 39, 41]. Interestingly, expressing mutant BICD2 in *Drosophila* muscles has no obvious effect on motor function, while neuron-specific expression resulted in reduced neuromuscular junction size in larvae and impaired locomotion of adult flies [30]. Combined with the observation that mutant BICD2 causes axonal aberrations and increased microtubule stability in motor neurons points to a neurological cause of the disease [30]. More recently, a p.Arg694Cys (R694C) mutation in the C-terminal CC3 RAB6/RANBP2-binding domain of BICD2 was found to be associated with severe neuromuscular defects, but also disordered cortical development with in utero onset [43]. This disease has been classified as the neuronal disorder SMALED2B (OMIM#618291) [53]. As such, BICD2 seems associated with human malformations in cortical developments such as polymicrogyria (PMG), and the spectrum of BICD2-associated malformations overlaps with the wide spectrum of developmental abnormalities found in patients with DYNC1H1 mutations [11]. This leads to the speculation that BICD2 might play a different role in dynein-mediated processes in different brain regions, as well as in mitotic versus post-mitotic cells. Although there is strong human genetic evidence that BICD2 plays an important role in the development of the nervous system, it is poorly understood which cellular and molecular function of BICD2 is altered in these patients, and particularly little is known about the role of BICD2 during cortical development. Since PMG is thought to be a late neuronal migration defect [25], we hypothesized a pivotal role for BICD2 in neuronal migration.

Previous studies have shown that in the mouse cerebellum, depletion of BICD2 leads to severe lamination defects. The migration of cerebellar neurons is entirely dependent on *Bicd2* expression in Bergmann glia cells, while *Bicd2* is not expressed in cerebellar neurons [24]. In the cortex, BICD2 knockdown by in utero electroporation (IUE) was reported to cause impaired neurogenesis and early migration defects. These defects, at least in part, were found to follow from disrupted INM and aberrant mitosis in RGPs [21]. However, RGPs in the cerebral cortex give rise to both neurons and glia cells, and also act as scaffolds for radial migration [40]. This makes it difficult to differentiate between potential glia- and neuron-specific defects, and to decipher to which extent defects in cortical organization follows from abnormal neurogenesis or from impaired cortical neuron migration.

To define the precise role of BICD2 during cortical development and in particular to dissect its specific function in excitatory neurons versus RGPs in vivo, we compared two conditional knock-out (cKO) mouse lines. Emx1-driven *Bicd2* cKO mice, which are BICD2-deficient in RGPs and post-mitotic neurons, were compared with Nex-driven *Bicd2* cKO mice, which are only BICD2-deficient in post-mitotic migrating neurons. We show that BICD2 is expressed in developing cortical neurons and that radial cortical migration and corticogenesis predominantly depends on BICD2 function in post-mitotic neurons. Neuron-specific BICD2-KO mice showed severely impaired radial migration of late-born upper-layer neurons, and single-neuron labeling revealed a specific role for BICD2 in bipolar locomotion during neuronal migration. BICD2 depletion in cortical neurons interfered with Golgi apparatus organization in the leading edges and caused apoptotic cell death of cortical plate neurons. Using rescue experiments with disease-related *Bicd2* mutations, we found that a specific mutation in the RAB6/RANBP2-binding-domain, which is associated with human cortical malformations, fails to restore proper cortical neuron migration. Together, these findings demonstrate a novel, cell-intrinsic role of BICD2 in cortical neuron migration in vivo and provide new insights into dynein-mediated functions during cortical development, and the role of dynein in cortical malformations.

## Results

### Neuronal migration and lamination in the cortex depend on the neuron-specific expression and function of BICD2 in excitatory neurons

To dissect the role of BICD2 in excitatory neurons versus RGPs during corticogenesis in vivo, we used two *Bicd2* cKO mouse lines. To generate *Bicd2*^*fl/fl*^*;Nex-Cre*^*+/−*^ mice (hereafter referred to as: Nex-KO), which are depleted of BICD2 exclusively in post-mitotic glutamatergic neurons of the cerebral cortex and the hippocampus, we crossed *Bicd2* floxed mice [24] with heterozygous Nex-Cre mice [13]. We compared these mice with *Bicd2*^*fl/fl*^*;Emx1-Cre*^*+/−*^ mice (hereafter referred to as: Emx1-KO), which are depleted of BICD2 in RGPs, glutamatergic neurons, and astrocytes in the cerebral cortex and the hippocampus, that were previously created by crossing homozygous *Bicd2* floxed mice with heterozygous Emx1-Cre mice [15, 24]. The homozygous floxed littermates (*Bicd2*^*fl/fl*^*;Emx1-Cre*^*−/−*^ mice and *Bicd2*^*fl/fl*^*;Nex-Cre*^*−/−*^ mice; hereafter referred to as Emx1-WT and Nex-WT) were used as controls. In contrast to the global *Bicd2* KO [24], the offspring of both cKO lines were born in Mendelian frequencies, viable and fertile (data not shown).

Analysis of BICD2 expression in E17.5 neocortices using immunohistochemistry showed that BICD2 staining was strongly reduced in Emx1-KO and Nex-KO cortices (Additional file 1: **Fig. S1a-c**) and hippocampi, while BICD2-immunoreactivity was present in control mice. No changes in BICD2 immunoreactivity were observed in other brain areas, such as the striatum (Additional file 1: **Fig. S1a**), consistent with the selectivity of Emx1-Cre and Nex-Cre for the dorsal telencephalon [13, 15]. Closer inspection of loss of BICD2-immunoreactivity in the cortex showed a consistent difference between Emx1-KO and Nex-KO mice: in Emx1-KO mice, BICD2 immunostaining was reduced in both the superficial and deep regions of the cortex (Additional file 1: **Fig. S1c**). In particular, immunoreactivity disappeared from the RGPs facing the ventricular border of the cortex (Additional file 1: **Fig. S1c**). In Nex-KO mice, BICD2-immunoreactivity was strongly reduced in superficial layers but not in deep cortical regions. In both Emx1-KO and Nex-KO mice, the cytosolic BICD2-immunoreactivity in post-mitotic neurons was strongly reduced. However, different from Emx1-KO mice but similar to control mice, Nex-KO mice showed BICD2-immunoreactivity in the cytosol of RGPs and increased punctate staining at the ventricular surface (Additional file 1: **Fig. S1c**). Together, these immunostainings show that in the cerebral cortex, unlike in the cerebellum where *Bicd2* is exclusively expressed in Bergmann glia cells [24], *Bicd2* is expressed in both RGPs and excitatory neurons. The substantial reduction of BICD2 in both Emx1-KO and Nex-KO cortices was confirmed by western blot analyses of whole cortex lysates with three different anti-BICD2 antibodies (Additional file 1: **Fig. S1d,e**).

**Fig. 1:**
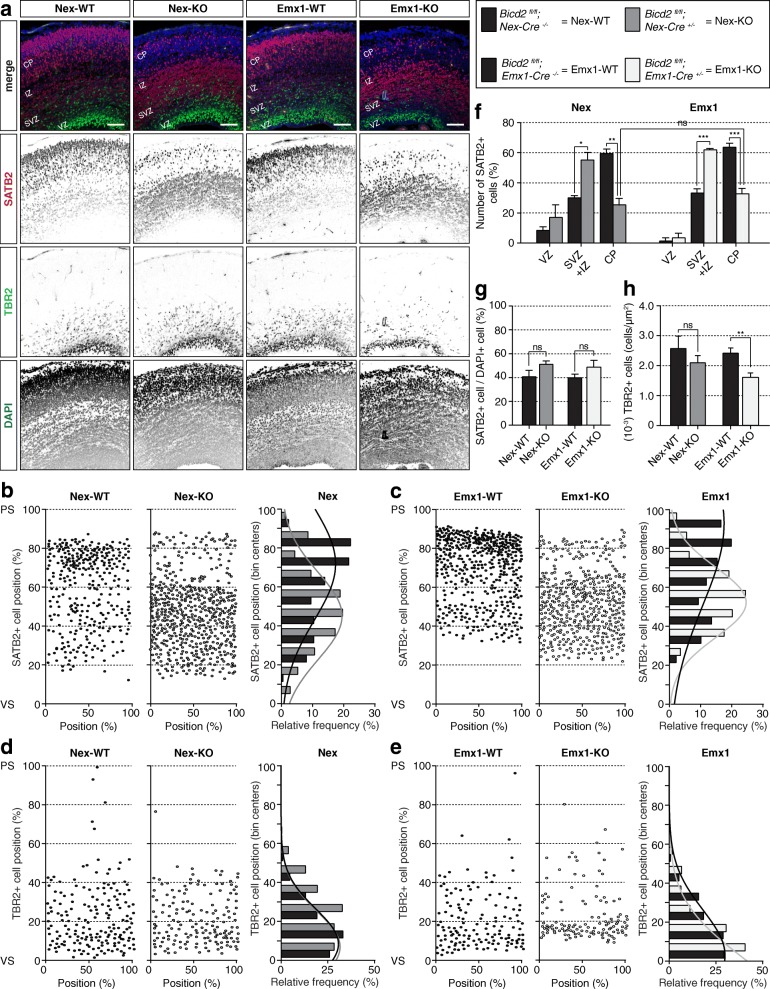
Neuronal migration and lamination in the cortex depends on the neuron-specific expression and function of BICD2 in excitatory radial migrating neurons. **a.** Coronal cryo-sections of E17.5 cortices from cell-type-specific conditional *Bicd2* KO mice and their control littermates - *Bicd2*^*fl/fl*^*;Nex-Cre*^*+/−*^ (=Nex-KO), *Bicd2*^*fl/fl*^*;Emx1-Cre*^*+/−*^ (=Emx1-KO), *Bicd2*^*fl/fl*^*;Nex-Cre*^*−/−*^ (=Nex-WT) and *Bicd2*^*fl/fl*^*;Emx1-Cre*^*−/−*^ (=Emx1-WT) respectively - were stained against the upper-layer (II/III) marker SATB2 (red) and the intermediate basal progenitor marker TBR2 (green). DAPI is shown in blue. Scale bars are 100 μm. **b + c.** Graphical representation of the relative position of SATB2+ cells over the cortical longitude from ventricular (VS) to pial surface (PS) and 187.5 μm in width (both in %) (left panels); and quantification of the relative frequency of SATB2+ cells over the cortical longitude (%, binned in centers) and their gaussian distribution (right panels) for Nex-WT and Nex-KO mice (**b**) and Emx1-WT and Emx1-KO mice (**c**). **d + e.** Graphical representation of the relative position of TBR2+ over the cortical longitude from VS to PS and 187.5 μm in width (both in %) (left panels); and quantification of the relative frequency of TBR2+ cells over the cortical longitude (%, binned in centers) and their gaussian distribution (right panels) for Nex-WT and Nex-KO mice (**d**) and Emx1-WT and Emx1-KO mice (**e**). **f.** Relative amount of SATB2+ cells in VZ, SVZ/IZ and CP. For SATB2+ cells, we have counted cell location for at least 3 mice (*N* = 3–6, average cell position per mice is represented as individual data points in the graph) for each genotype coming from at least 2 different litters. Between 237 and 640 cells have been counted per mouse (*n* = 237–640). **g.** Relative amount of SATB2+ cells in the cortex, based on ratio of SATB2+/DAPI+ cells. **h.** Number (10^− 3^) of TBR2+ cells per μm^2^ (*N* = 5–9, *n* = 150–464). CP: cortical plate, IZ: intermediate zone, PS: pial surface, SVZ: subventricular zone, VS: ventricular surface, VZ: ventricular zone. *** *p* < 0.001, ** *p* < 0.005, * *p* < 0.05, ns = not significant; error bars are ±SEM. Used tests: One Way ANOVA with Sidak’s multiple comparison (**f**), Mann-Whitney *U* test (**g, h**)

Further anatomical examination of the developing cerebral cortex revealed that the radial diameter of the cerebral cortex was reduced in both the Emx1-KO and Nex-KO mice (Additional file 1: **Fig. S1f**). Next, we mapped differences in the laminar organization of the cortex at E17.5 using multiple markers. At this stage, most cortical projection neurons have nearly completed their radial migration into the cortical plate (CP) and are defined by layer-specific transcription factors. Immunostaining against SATB2, a transient marker of post-mitotic cortical excitatory neurons that predominantly labels the layer II/III neurons [1, 4], showed that in Emx1-WT mice, most (~ 60%) SATB2+ neurons reached the upper layers of the CP. In Emx1-KO mice however, the SATB2+ neurons failed to migrate towards the upper layers of the CP and accumulated in the intermediate zone (IZ) and SVZ (Fig. 1a,c).

To determine the role of BICD2 specifically in post-mitotic neurons, we compared this with neuronal migration in Nex-KO mice. We found comparable migration defects in the Nex-KO (Fig. 1a,b), with most SATB2+ cells located in the IZ/SVZ instead of the CP. The majority (~ 60%) of SATB2+ neurons had migrated to the upper layers of the CP in Nex-WT littermates. The increased percentage of SATB2+ neurons in the IZ/SVZ and the decreased percentage of neurons migrated into the CP were comparable in Nex-KO and Emx1-KO mice (55.90 ± 4.82 in the SVZ/IZ of Nex-KO mice versus 62.38 ± 1.86 in Emx1-KO mice versus and 26.11 ± 3.81 in the CP of Nex-KO versus 33.45 ± 3.00 in Emx1-KO mice, Fig. 1f). This suggests that proper neuronal migration in the cortex in vivo does not primarily depend on BICD2 function in RGPs or glia cells, but rather on the cell-intrinsic function of BICD2 in post-mitotic radially migrating neurons. The total number of SATB2+ cells over the ventricular-to-pial extent was unaltered in both Nex-KO and Emx1-KO cortices at E17.5 (Fig. 1g), even though the number of TBR2+ intermediate basal progenitor cells was reduced in Emx1-KO, but not in the Nex-KO (Fig. 1h). The relative position of TBR2+ intermediate basal progenitor cells was not altered in the cortex of both Nex-KO and Emx1-KO mice (Fig. 1d,e). These data suggest that BICD2 mainly regulates migration, and not neurogenesis, of late-born upper-layer neurons.

### BICD2 is essential for radial migration of upper-layer neurons, but not for the migration of deeper-layer neurons

**Fig. 2:**
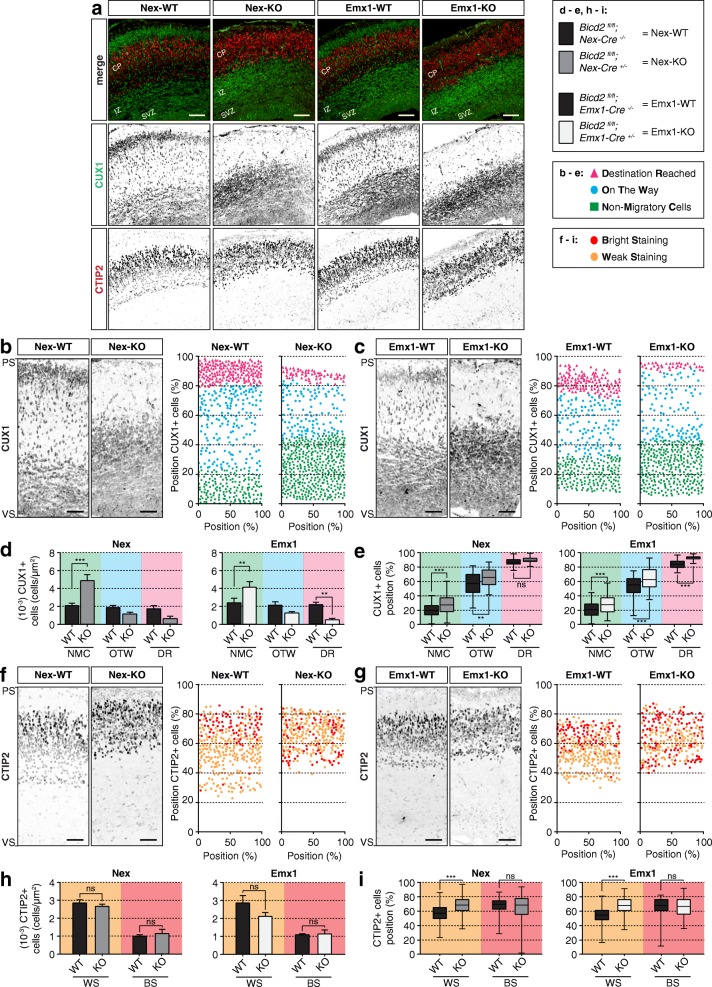
BICD2 is essential for radial migration of upper-layer neurons but not for the migration of deeper-layer neurons. **a.** Coronal cryo-sections of E17.5 cortices from cell-type-specific conditional *Bicd2* KO mice and their control littermates - *Bicd2*^*fl/fl*^*;Nex-Cre*^*+/−*^ (=Nex-KO), *Bicd2*^*fl/fl*^*;Emx1-Cre*^*+/−*^ (=Emx1-KO), *Bicd2*^*fl/fl*^*;Nex-Cre*^*−/−*^ (=Nex-WT) and *Bicd2*^*fl/fl*^*;Emx1-Cre*^*−/−*^ (=Emx1-WT) respectively - were stained against the deeper cortical layer (V/VI) marker CTIP2 (red) and the superficial layer marker CUX1 (green). Scale bars are 100 μm. **b + c.** Selected areas from ventricular (VS) to pial surface (PS) and 156.3 μm width (left panels) and graphical representation of the relative position of CUX1+ cells over the cortical longitude from VS to PS (in %) and 156.3 μm in width (in %) which did not migrate (Non-Migratory Cells = green), were still migrating (On The Way = blue) and reached cortical layer II/III (Destination Reached = pink) (right panels) for Nex-WT and Nex-KO mice (**b**) and Emx1-WT and Emx1-KO mice (**c**). Scale bars are 50 μm. **d + e.** Number (10^− 3^) of CUX1+ cells per μm^2^ (**d**) and their distribution cells as relative position over the cortical longitude from VS to PS (in %) (**e**) which did not migrate (NMC: Non-Migratory Cells = green), were still migrating (OTW: On The Way = blue) and reached cortical layer II/III (DR: Destination Reached = pink) for Nex-WT and Nex-KO mice (left) and Emx1-WT and Emx1-KO mice (right) (*N* = 3–4, *n* = 389–611). **f + g.** Selected area from VS to PS and 156.3 μm width (left panels). Layer VI neurons show weak CTIP2 staining (light gray) and layer V neurons bright CTIP2 staining (dark gray). Scale bars are 50 μm. Right panels are graphical representations of the relative position of CTIP2+ layer VI neurons with weak CTIP2 staining (WS, yellow) and layer V neurons with bright CTIP2 staining (BS, red) over the cortical longitude from VS to PS (in %) and 156.3 μm in width (in %) for Nex-WT and Nex-KO mice (**f**) and Emx1-WT and Emx1-KO mice (**g**). **h + i.** Number (10^− 3^) of CTIP2+ cells per μm^2^ (**h**) and their distribution cells as relative position over the cortical longitude from VS to PS (in %) (**i**) with weak CTIP2 staining (WS, yellow) and bright CTIP2 staining (BS, red) for Nex-WT and Nex-KO mice (left) and Emx1-WT and Emx1-KO mice (right) (*N* = 3–4, *n* = 232–398). CP: cortical plate, IZ: intermediate zone, PS: pial surface, SVZ: subventricular zone, VS: ventricular surface. *** *p* < 0.001, ** *p* < 0.005, * *p* < 0.05, ns = not significant; error bars are ±SEM. Used tests: One-Way ANOVA with Sidak’s multiple comparisons (**d, h**), Kruskal Wallis test with Dunn’s multiple comparisons (**e, i**)

To characterize the lamination defects in more detail, we further analyzed neuronal migration by labeling for CUX1, which is a marker for superficial layer neurons [33, 36], and CTIP2, a marker for layer V/VI neurons [2, 33]. Similar to SATB2+ neurons, the late-born CUX1+ neurons in Emx1- and Nex-KO mice failed to migrate into the CP and accumulated in the SVZ and IZ (Fig. 2a-c). At E17.5, the migration of CUX1+ layer II/III neurons is not yet completed [36], and accordingly, we observe that only part of the CUX1+ cells have accumulated in the superficial part of the CP representing their final destination, while many cells are distributed throughout deeper regions of the CP, as well as in the IZ, SVZ and VZ, providing a snapshot of neurons before, during and after radial migration (Fig. 2b). The prominent band observed in Nex-WT and Emx1-WT of brightly CUX1-labeled neurons in the upper CP, representing neurons after radial migration, was nearly absent in Emx1-KO and Nex-KO mice (Fig. 2b). Most CUX1+ neurons showed impaired migration, and accumulated below the CP in both cKOs (Fig. 2d).

Earlier born deeper-layer CTIP2+ neurons appeared much less affected in their migration than upper-layer neurons and many could be observed in the CP. Prospective layer VI neurons were marked by weak CTIP2 immunostaining and their location was not affected in Nex-KO (Fig. 2f) and Emx1-KO (Fig. 2g) mice. Prospective layer V neurons were marked by bright CTIP2 immunostaining and localized above layer VI in Nex-WT and Emx1-WT littermates. In both cKOs, CTIP2+ cells seemingly all populated the CP, reminiscent of the distribution of CTIP2+ cells in control. However, the bright and weakly labeled CTIP2+ cells were not concentrated in two distinct layers. Instead, the prospective layer V neurons largely overlapped with prospective layer VI neurons (Fig. 2f,g,i), suggesting that their migration was slightly impaired. The number of CTIP2+ cells was not altered in cKO mice (Fig. 2h). Notably, the early-born CTIP2 expressing neurons in cKO cortices were found to localize at higher relative positions (more apical), defined as relative distance from the ventricular surface (VS; basal) (Fig. 2i), and apical instead of basal to CUX1+ neurons (Fig. 2a). The altered distributions of CTIP2+ and CUX1+ cells could either point to a global inversion of cortical layers in Nex-KO and Emx1-KO mice, or be the consequence of impaired layer II/III neuron migration.

First-born TBR1+ layer VI neurons did not show radial migration defects in Nex-KO (Additional file 2: **Fig. S2a,d**) and Emx1-KO mice (Additional file 2: **Fig. S2a,e)** and formed the first layer of the CP just above the IZ (Additional file 2: **Fig. S2a,f**). Similar to CTIP2+ neurons, TBR1+ neurons were localized at more apical positions in cKO cortices, because the diameter of the CP was reduced (Additional file 2: **Fig. S2f**). The upper SVZ and IZ, which contained the upper-layer neurons in *Bicd2* deficient mice, were markedly thicker and less well organized (Additional file 2: Fig. 2a**,S2a**). Both cKO mice lacked a well-restricted, low-somata-density IZ containing the well-bundled axons of contra-lateral and cortico-fugal projecting axons (Additional file 2: **Fig. S2a**): while the neurofilament heavy chain (NF) labeled axons formed well-organized bundles running in a narrow band in the IZ in control littermates, the axonal tracts in Emx1-KO and Nex-KO mice were much less organized and instead of running bundled in a restricted band, spread out over the cortical longitude (Additional file 2: **Fig. S2a-c**). Importantly, the number of first-born TBR1+ neurons at E17.5 was unaltered in both Nex-KO and Emx1-KO cortices (Additional file 2: **Fig. S2g**). These data support the idea that BICD2 has an essential cell-intrinsic role during cortical neuron migration in vivo specific for upper-layer neurons.

BICD2-depletion had a seemingly stronger impact on the organization of contra-lateral projecting NF+ axons than on the organization of radial RGP-processes: immunostaining against Nestin revealed that the radial orientation of the RGP-processes was not impaired in Nex-KO. The radial organization of Nestin+ fibers was also not disturbed in Emx1-KO, even if the total amount of fibers appeared reduced and the basal RGP-processes showed a slightly abnormal pattern (Additional file 2: **Fig. S2h**). The nearly unaffected RGPs organization in Nex-KO mice suggests that the disorganization of axonal bundles in the IZ is indeed a result of BICD2 loss in neurons, and independent of *Bicd2* expression in RGPs.

### BICD2 is required for Golgi organization and integrity in the cortical plate

BICD2 is known to be important for Golgi integrity and overexpression of disease-related point-mutant forms of BICD2 leads to Golgi fragmentation [31, 41]. However, the impact of BICD2 on Golgi integrity in developing cortical neurons is unknown. Morphological changes of Golgi apparatus of CP neurons in Emx1-KO and Nex-KO mice were observed. GM130-stained trans-Golgi was found in control mice as a compact structure close to the nucleus in most VZ, SVZ and IZ located cells. In the CP however, the Golgi was organized as long, continuous stretches in radial orientation (Additional file 2: **Fig. S3**). In cells located directly at the VS we detected similar long, radial Golgi-stretches, in agreement with previous reports where the trans-Golgi was detected in the apical processes of RGPs [58]. In contrast, the radial Golgi-stretches in the CP showed a disrupted and discontinuous pattern and fail to organize in long, continuous stretches in both Emx1-KO and Nex-KO (Additional file 2: **Fig. S3c,d**). These results suggest that BICD2 might play a role in Golgi organization of neurons in the CP and elongation of the trans-Golgi into the neuronal leading edges.

### BICD2 is required for nuclear migration in upper-layer cortical neurons during locomotion mode

**Fig. 3:**
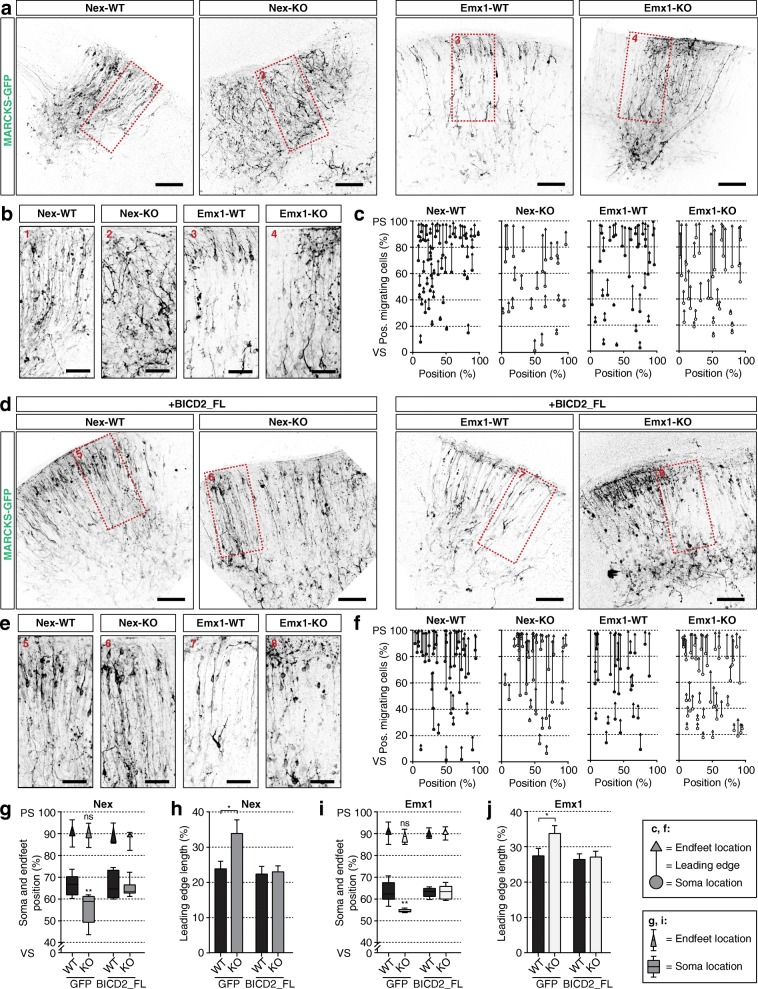
BICD2 is required for nuclear migration in upper-layer cortical neurons in locomotion mode. Ex vivo brain electroporation with MARCKS-GFP at E14.5, followed by organotypical slice cultures for 4 DIV of cell-type-specific conditional *Bicd2* KO mice and their control littermates - *Bicd2*^*fl/fl*^*;Nex-Cre*^*+/−*^ (=Nex-KO), *Bicd2*^*fl/fl*^*;Emx1-Cre*^*+/−*^ (=Emx1-KO), *Bicd2*^*fl/fl*^*;Nex-Cre*^*−/−*^ (=Nex-WT) and *Bicd2*^*fl/fl*^*;Emx1-Cre*^*−/−*^ (=Emx1-WT) - respectively. **a**. Organotypical coronal slices of Nex-WT, Nex-KO, Emx1-WT and Emx1-KO mice at E14.5 + 4 DIV. Cells are labeled with MARCKS-GFP through ex vivo electroporation. Scale bars are 100 μm. **b**. Selected areas from images shown in (**a**). Scale bars are 50 μm. **c.** Graphical representation of the relative positions of GFP+ soma (circles), leading edges (lines) and endfeet (triangles) over the cortical longitude from ventricular (VS) to pial surface (PS) (in %) and 156.3 μm in width (in %). **d**. Organotypical coronal slices of Nex-WT, Nex-KO, Emx1-WT and Emx1-KO mice at E14.5+ 4 DIV. Cells are transfected with full-length GFP-BICD2 (BICD2_FL) and MARCKS-GFP through ex vivo electroporation. Scale bars are 100 μm. **e.** Selected areas from images shown in (**d**). Scale bars are 50 μm. **f**. Graphical representation of the relative positions of GFP+ soma (circles), leading edges (lines) and endfeet (triangles) over the cortical longitude from ventricular (VS) to pial surface (PS) (in %) and 156.3 μm in width (in %). **g + i.** Relative position of GFP+ soma (squares) and endfeet (triangles) over the cortical longitude from VS to PS for Nex-WT and Nex-KO (**g**) (*N* = 5–8, *n* = 21–148), and Emx1-WT and Emx1-KO mice (**i**) (*N* = 4–11, *n* = 29–151). **h + j**. Average length of the leading edges in % of total cortical radial diameter from VS to PS for Nex-WT and Nex-KO (**h**), and Emx1-WT and Emx1-KO mice (**j**). PS: pial surface, VS: ventricular surface. ** *p* < 0.005, * *p* < 0.05, ns = not significant; error bars are ±SEM. Used tests: One Way ANOVA with Dunnet’s multiple comparisons (**g, h, i, j**)

To dissect which steps and which cellular processes in radial migration are affected and causing the observed deficits in Nex-KO and Emx1-KO mice, we performed ex vivo brain electroporations (EVE) to visualize the morphology of individual migrating neurons. We labeled nascent neurons at E14.5 with MARCKS-GFP and analyzed fluorescently-labeled neurons after 4 days of organotypical cortical slice cultures. In control mice slices, the majority of labeled neurons had acquired a bipolar morphology with one leading edge reaching the pia and one trailing axon (Fig. 3a-c). Neuronal soma were present in the upper CP, with a short leading edge and a long axon. Similarly, in Nex-KO and Emx1-KO slices, the majority of the labeled cortical neurons showed bipolar cell morphology with one dominant leading edge elongating until the pial surface (Fig. 3a,b). While leading edge extension and endfeet location were the same as in control (Fig. 3c,g,i), their soma were found at more basal positions (Fig. 3g,i). As a consequence of this basal position of the soma and thus nuclei, the leading edges of these cells were longer in Nex-KO and Emx1-KO mice (Fig. 3h,j). To validate overall tissue integrity DAPI staining was performed; no obvious defects or differences are observed with previously shown histological tissue samples (data not shown).

To validate the role of BICD2 in bipolar radial migration, we performed a rescue experiment by expressing a full-length GFP-BICD2 (BICD2_FL) construct. This fully rescued the observed phenotypes in slices of both cKO mice (Fig. 3d,e**)**: neuronal soma were localized to comparable positions as in control mice (Fig. 3f,g,i), suggesting a restoration of nuclear migration. Likewise, average leading edge length was restored to normal in cKO situations following BICD2_FL overexpression (Fig. 3h,j). Notably, overexpression of BICD2_FL had no dominant effect on the neuronal migration in wildtype mice. Rescue of neuronal migration defects in Nex-KO with BICD2-FL confirms the cell-intrinsic function of BICD2 in radial neuron migration.

To further address the nuclear migration defects we detected in the EVE analyses, we also visualized the morphology of individual migrating neurons in the neuron-specific Nex-KO and control littermates by placing DiI crystals in the IZ of lightly-fixed cortical brain sections from E17.5 mice. At this stage of embryonic development, radial locomotion was nearly completed in control mice. Comparable to migration after 4 days in slice culture, the leading edge endfeet of the labeled neurons had reached the MZ and the nuclei were located in upper cortical layers, resulting in a bipolar morphology with a short leading edge and long axons (Additional file 4: **Fig. S4**). In Nex-KO mice, most labeled neurons displayed a bipolar morphology with a single axon and one radial leading edge. Although most leading edges of bipolar neurons in Nex-KO mice nearly reached the MZ, their nuclei were located at more basal positions in the CP compared to control mice (Additional file 4: **Fig. S4**). Consistent with the EVE experiments, the DiI-labeled neurons in Nex-KO mice seem to have elongated leading edges. Together, these results suggest that BICD2 plays a specific role in radial locomotion of cortical neurons by mediating nuclear migration in these neurons.

### *Bicd2* mutation at R694C, associated with human cortical malformation, impairs neuronal migration and nuclear migration in conditional *Bicd2* KO mice

**Fig. 4:**
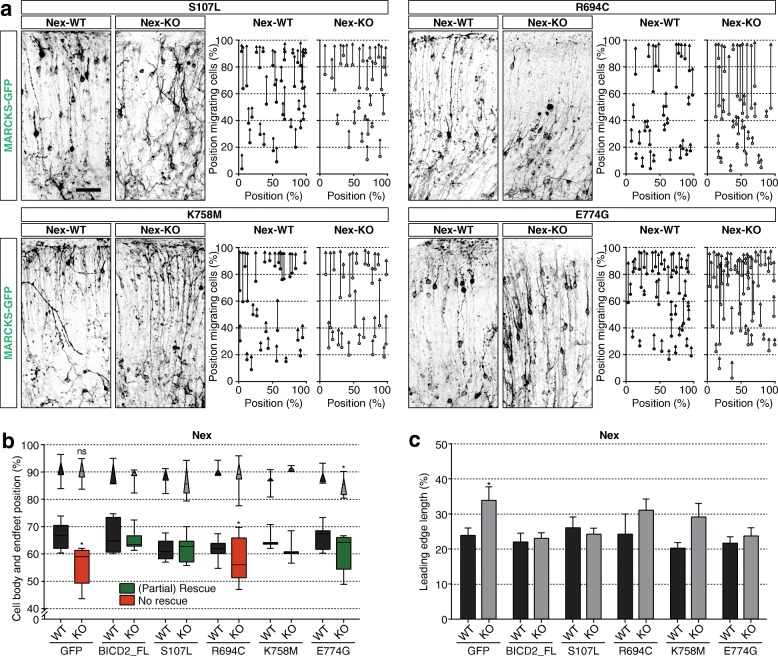
The SMALED2B-associated *Bicd2* point mutation fails to rescue neuronal migration defects. Ex vivo brain electroporation with MARCKS-GFP and BICD2 point mutants (GFP-BICD2_S107L (S107 L), GFP-BICD2_R694C (R694C), GFP-BICD2_K758M (K758M), GFP-BICD2_E774G (E774G)), at E14.5, followed by organotypical slice cultures for 4 DIV of cell-type-specific conditional *Bicd2* KO mice and their control littermates - *Bicd2*^*fl/fl*^*;Nex-Cre*^*+/−*^ (=Nex-KO) and *Bicd2*^*fl/fl*^*;Nex-Cre*^*−/−*^ (=Nex-WT). **a**. Selected zooms of organotypical coronal slices, and corresponding relative position of GFP+ soma (squares) and endfeet (triangles) over the cortical longitude from ventricular to pial surface, of Nex-WT and Nex-KO at E14.5 + 4 DIV transfected with MARCKS-GFP and indicated BICD2 point mutants. Scale bars are 50 μm. **b.** Relative position of GFP+ soma (squares) and endfeet (triangles) over the cortical longitude from ventricular to pial surface for Nex-WT and Nex-KO transfected with indicated BICD2 point mutants (*N* = 3–10, *n* = 6–197). **c**. Average length of the leading edges in % of total cortical radial diameter from ventricular to pial surface for Nex-WT and Nex-KO transfected with indicated BICD2 point mutants (*N* = 3–10, *n* = 6–197).* *p* < 0.05, ns = not significant; error bars are ±SEM. Used tests: One Way ANOVA with Dunnet’s multiple comparisons (**b** (cell bodies), **c**), Kruskal Wallis test with Dunn’s multiple comparisons (**b** (endfeet))

Point mutations in BICD2 have been found in patients with neuronal diseases such as SMALED2A and SMALED2B [53]. Which cellular and molecular function of BICD2 is altered in these patients, is poorly understood, and how phenotypic variation is caused by different point mutations still has to be elucidated. To address the cell-intrinsic cellular and molecular function of specific BICD2 domains in cortical neuron migration in vivo*,* we expressed different BICD2 point-mutations in the Nex-KO background. We electroporated the brains of Nex-KO and their control littermates with MARCKS-GFP in combination with the SMALED2B BICD2_R694C mutation, the SMALED2A mutations BICD2_S107L and BICD2_E774G, or *Drosophila* lethal BICD2_K758M and determined the rescue capabilities of the knockout migration phenotype. The SMALED2A and *Drosophila* lethal mutants partially or fully rescued neuronal migration defects of Nex-KO mice, with soma localizing higher in the cortex at similar positions to Nex-WT or BICD2_FL rescue (Fig. 4a), endfeet close to the pial surface (Fig. 4b) and unchanged leading edge lengths (Fig. 4c). The SMALED2B related BICD2_R694C was the only point mutant unable to rescue neuronal migration defects (Fig. 4a): neuronal soma generally failed to reach the upper layers of the cortex, and localized at similar positions as in Nex-KO transfected with MARCKS-GFP (Fig. 4b). The location of the endfeet was unchanged, and most reach the pial surface, resulting in slightly elongated leading edges (Fig. 4c). In summary, the cortical malformation-associated mutation R694C does not rescue radial migration defects observed in neuron-specific *Bicd2* knockout mice.

### Depletion of BICD2 causes neuronal cell death and affects neuronal maturation

**Fig. 5:**
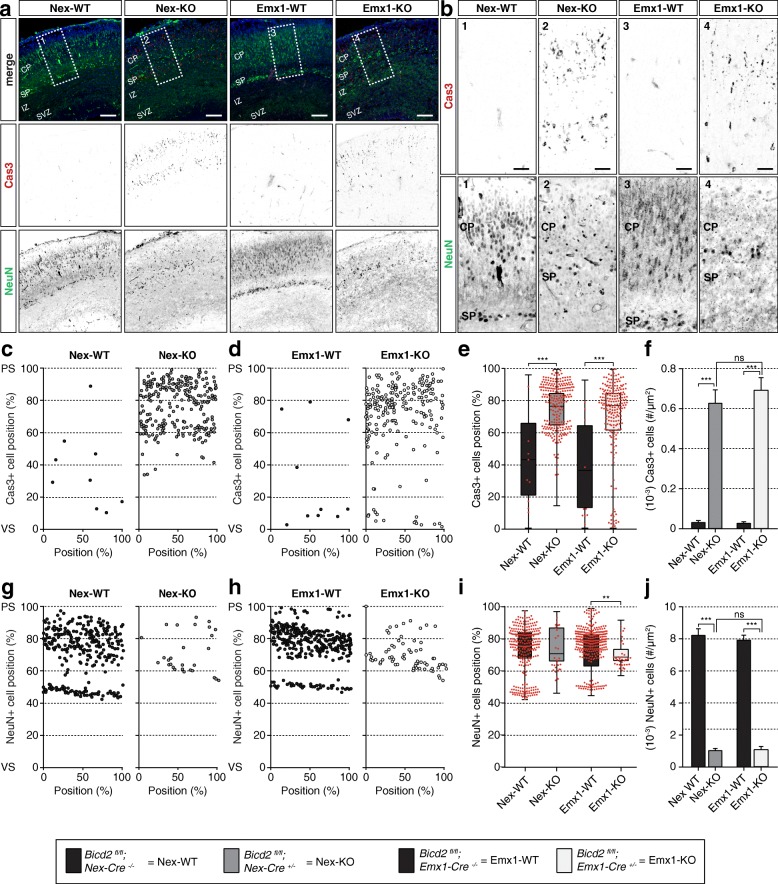
Depletion of BICD2 in the cortex impairs neuronal maturation and survival. **a.** Coronal cryo-sections of E17.5 cortices from cell-type-specific conditional *Bicd2* KO mice and their control littermates - *Bicd2*^*fl/fl*^*;Nex-Cre*^*+/−*^ (=Nex-KO), *Bicd2*^*fl/fl*^*;Emx1-Cre*^*+/−*^ (=Emx1-KO), *Bicd2*^*fl/fl*^*;Nex-Cre*^*−/−*^ (=Nex-WT) and *Bicd2*^*fl/fl*^*;Emx1-Cre*^*−/−*^ (=Emx1-WT) respectively - were stained against cleaved Caspase-3 (Cas3, red) as marker for apoptotic cell death and NeuN (green) as marker for mature neurons. Scale bars are 100 μm. **b.** Zoom of selected areas indicated in (**a**) show the Cas3 (top panels) and NeuN (lower panels) immunostaining in the CP. **c + d.** Graphical representation of the relative position of Cas3+ cells over the cortical longitude from ventricular (VS) to pial surface (PS) and 156.3 μm in width (both in %) in Nex-WT and Nex-KO (**c**) and Emx1-WT and Emx1-KO (**d**) cortices. **e.** Distribution and accumulated number of Cas3+ cells from 3 experiments as relative position over the cortical longitude from VS to PS (in %). Red circles are individual Cas3+ cell locations of representative samples (*N* = 5–9, *n* = 7–282). **f.** Number (10^− 3^) of Cas3+ cells per μm^2^ (*N* = 5–9, *n* = 7–282). **g + h.** Graphical representation of the relative position of NeuN+ cells over the cortical longitude from ventricular (VS) to pial surface (PS) and 156.3 μm in width (both in %) in Nex-WT and Nex-KO (**c**) and Emx1-WT and Emx1-KO (**d**) cortices. **i.** Distribution and accumulated number of NeuN+ cells from 3 experiments as relative position over the cortical longitude from VS to PS (in %). Red circles are individual NeuN+ cell locations of representative samples (*N* = 3, *n* = 28–300). **j.** Number of NeuN+ cells per μm^2^ (*N* = 3, *n* = 28–300). CP: cortical plate, IZ: intermediate zone, PS: pial surface, SP: subplate, SVZ: subventricular zone, VS: ventricular surface. *** *p* < 0.001, ** *p* < 0.005, ns = not significant; error bars are ±SEM. used tests: Kruskal Wallis test with Dunn’s multiple comparisons (**e, i**), One Way ANOVA with Tukey’s multiple comparisons (**f, j**)

To address if other cellular processes besides neuronal migration are affected by depletion of BICD2 and influence cortical development in vivo, we also decided to look into the maturation and survival of neurons in the developing cortex of *Bicd2* cKO mice. Immunostaining against NeuN, which is a marker for maturing neurons, showed that at late stages of embryonic development, the number of NeuN+ neurons in the CP and in the subplate (SP) was strongly reduced in both Emx1-KO and Nex-KO mice (Fig. 5a,b,j). We also observed a dim and diffuse NeuN signal in the upper SVZ and IZ of Emx1-KO and Nex-KO mice, but not in control littermates. To address the survival of neurons during cortical development, we stained against cleaved Caspase-3 (Cas3), a marker for apoptosis. This revealed massive apoptotic cell death in cortices of both cKO mice (Fig. 5a,f), and notably, the apoptotic neurons were not observed in the IZ where the BICD2-deficient upper-layer neurons accumulate, but specifically in the SP and in the CP (Fig. 5c-e). In Emx1-KO cortices, we found a small additional population of Cas3+ cells in the VZ (Fig. 5b-d). To confirm that the depletion of BICD2 in the developing cortex causes specific apoptosis of maturing neurons in the CP, we compared these results with the in vivo situation at early stages of cortical development. At E14.5, we found nearly no apoptotic cells in Emx1-WT (Additional file 5: **Fig. S5a,b**) or Nex-KO mice (data not shown). In Emx1-KO E14.5 mice, the number of Cas3+ cells was significantly increased, but we found markedly less apoptotic cells (0.25 ± 0.02 *10^− 3^/μm^2^) than at E17.5 (0.70 ± 0.06 *10^− 3^/μm^2^) (Additional file 5: Fig. 5f**, S5c,d**). The apoptotic cells observed in Emx1-KO E14.5 cortices did not accumulate in the developing CP where the post-mitotic doublecortin (DCX) + neurons were found, but were spread over the entire cortex (Additional file 5: **Fig. S5c,e**). Together, these results suggest that the reduced number of mature NeuN+ neurons in *Bicd2* deficient cortices in vivo is not only the result of impaired neurogenesis [21], but might be due to a delay in neuronal maturation. In addition, the remarkable apoptotic cell death of cortical neurons in the CP suggests that not only maturation, but also neuronal survival is affected. The comparable number of apoptotic cells in Nex-KO and Emx1-KO mice (Fig. 5f) demonstrates that the cell death is caused by the loss of BICD2 in neurons and does not depend on *Bicd2* expression in RGPs.

### Depletion of BICD2 in RGPs in vivo does not decrease RGP division but alters the position and cell-cycle progression of dividing progenitor cells

**Fig. 6:**
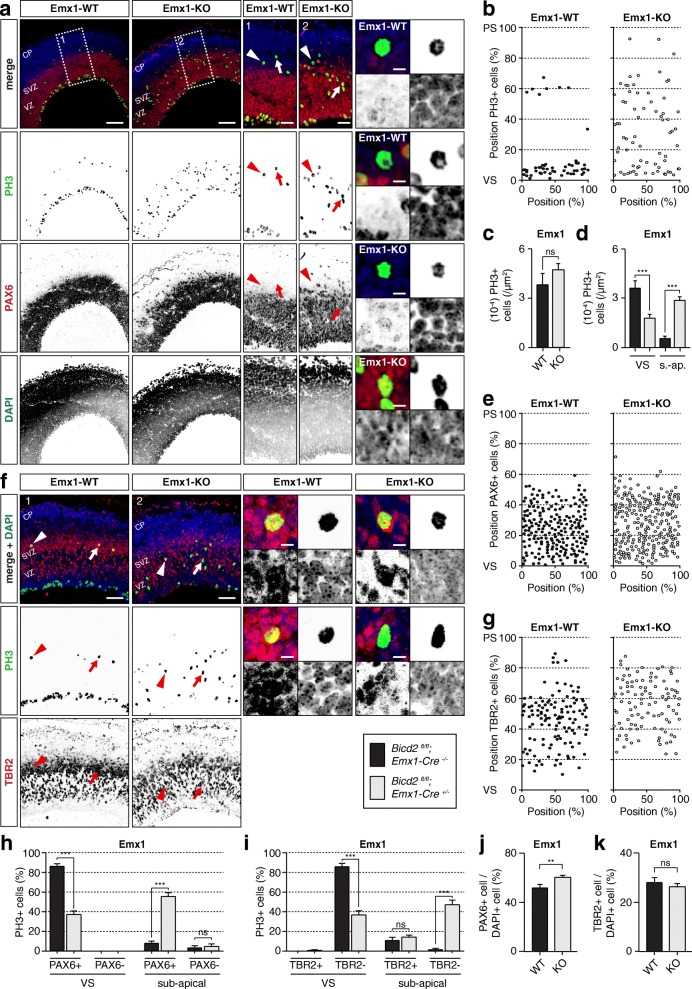
Depletion of BICD2 in RGPs in vivo does not decrease RGP division but alters the position and cell-cycle progression of dividing progenitor cells. **a.** Coronal cryo-sections of E14.5 cortices from *Bicd2*^*fl/fl*^*;Emx1-Cre*^*+/−*^ (=Emx1-KO) and *Bicd2*^*fl/fl*^*;Emx1-Cre*^*−/−*^ (=Emx1-WT) mice were stained against the apical progenitor marker PAX6 (red) and the RGP proliferation marker Phospho-Histone H3 (PH3) marker (green). DAPI is shown in blue. Scale bars in left panels are 100 μm; and 50 μm in the zooms. **b.** Graphical representation of the relative position of PH3+ cells over the cortical longitude from ventricular (VS) to pial surface (PS) (in %). **c**. Number (10^− 4^) of PH3+ cells per μm^2^ (*N* = 9–12, *n* = 20–72). **d**. Number (10^− 4^) of PH3+ cells per μm^2^ at the VS and sub-apical locations (*N* = 9–12, *n* = 20–72). **e.** Graphical representation of the relative position of PAX6 + cells over the cortical longitude from VS to PS (in %). **f.** Coronal cryo-sections of E14.5 cortices from Emx1-KO and Emx1-WT mice were stained against the basal intermediate progenitor marker TBR2 (red) and PH3 (green). DAPI is shown in blue. Scale bars in left panels are 100 μm; and 50 μm in the zooms. **g.** Graphical representation of the relative position of TBR2+ cells over the cortical longitude from VS to PS (in %). **h.** Relative amount of PH3+ cells positive or negative for PAX6 which divide at the VS or at sub-apical positions in the upper VZ/SVZ (*N* = 4–7, *n* = 20–72). **i.** Relative amount of PH3+ cells positive or negative for TBR2 which divide at the VS or at sub-apical positions in the upper VZ/SVZ (*N* = 4–6, *n* = 40–67). **j.** Number of PAX6+ cells as a ratio of the number of DAPI+ cells (*N* = 4–7, *n* = 20–72). **k**. Number of TBR2+ cells as a ratio of the number of DAPI+ cells (*N* = 4–6, *n* = 40–67). CP: cortical plate, PS: pial surface, SVZ: subventricular zone, VS: ventricular surface, VZ: ventricular zone. s.-ap: sub-apical. *** *p* < 0.001, ** *p* < 0.005, ns = not significant; error bars are ±SEM. Used tests: Unpaired t-test (**c, k**), One Way ANOVA with Tukey’s multiple comparisons (**d**), One Way ANOVA with Sidak’s multiple comparisons (**h, i**), Mann Whitney *U* test (**j**)

The previously shown defects in RGP divisions and neurogenesis by blocking apical nuclear migration following *Bicd2* knockdown [21], made us anticipate that Emx1-KO mice may show altered mitosis and neurogenesis, while Nex-KO mice do not. This could also explain the reduced levels of TBR2+ cells we observed in E17.5 Emx1-KO cortices compared to control and Nex-KO (Fig. 1). To dissect the potential function of BICD2 in cortical neurogenesis, we analyzed RGP proliferation and differentiation at E14.5 in Emx1-driven KO mice. Using Phospho-Histone 3 (PH3) as a marker for dividing cells, we found in control mice - as well as in the Nex-KO (data not shown) - most PH3+ dividing RGPs at the VS (Fig. 6a,b,d). In Emx1-KO mice however, the number of PH3+ cells dividing at the VS was significantly reduced (1.85 ± 0.19 * 10^− 4^/μm^2^ in Emx1-KO compared to 3.67 ± 0.40 * 10^− 4^/μm^2^ in control littermates) (Fig. 6d). While this reduced progenitor division at the VS in Emx1-KO mice in vivo is consistent with the knockdown of *Bicd2* by IUE [21], we observed that Emx1-driven depletion of BICD2 in RGPs in vivo drastically enhanced the number of PH3+ cells at ectopic sub-apical positions (outer VZ and SVZ) (Fig. 6b,d). This was not observed for Nex-KO mice (data not shown). Due to the massive increase of PH3+ progenitors dividing at an ectopic position, the total number of PH3+ mitotic cells was not decreased in the Emx1-KO compared to control mice (Fig. 6c). In rodent neurogenesis, the sequential transition from RGP, also known as apical progenitors (AP), to iBP to post-mitotic neuron is correlated with the sequential expression of the transcription factors PAX6, TBR2, and TBR1 [10]. While APs dividing apically at the VS are known to express PAX6, iBPs dividing in the SVZ are TBR2+. To determine if the PH3+ cells at ectopic sub-apical positions in Emx1-KO mice are still PAX6+ RGPs dividing at ectopic positions, or already committed to an iBP cell fate and positive for TBR2, we co-immunostained against PH3 and PAX6. In Emx1-KO all PH3+ cells which are still located at the VS were PAX6+, but also nearly all of the additional PH3+ at sub-apical positions were still positive for the AP marker PAX6 (**arrow** Fig. 6a**,** Fig. 6h). Accordingly, all PH3+ cells that remained at the VS were negative for TBR2, and the majority of the additional sub-apical PH3+ cells showed no staining for the iBP marker TBR2 (**arrow** Fig. 6f**,** Fig. 6i). In fact, the percentage of PH3+/TBR2+ double-labeled cells at sub-apical positions in Emx1-KO cortices did not exceed the percentage of double-labeled cells in Emx1-WT mice (Fig. 6i), illustrating that all additional ectopic PH3+ cells dividing at sub-apical positions in Emx1-KO mice were indeed PAX6+ APs but not TBR2+ iBPs.

**Fig. 7:**
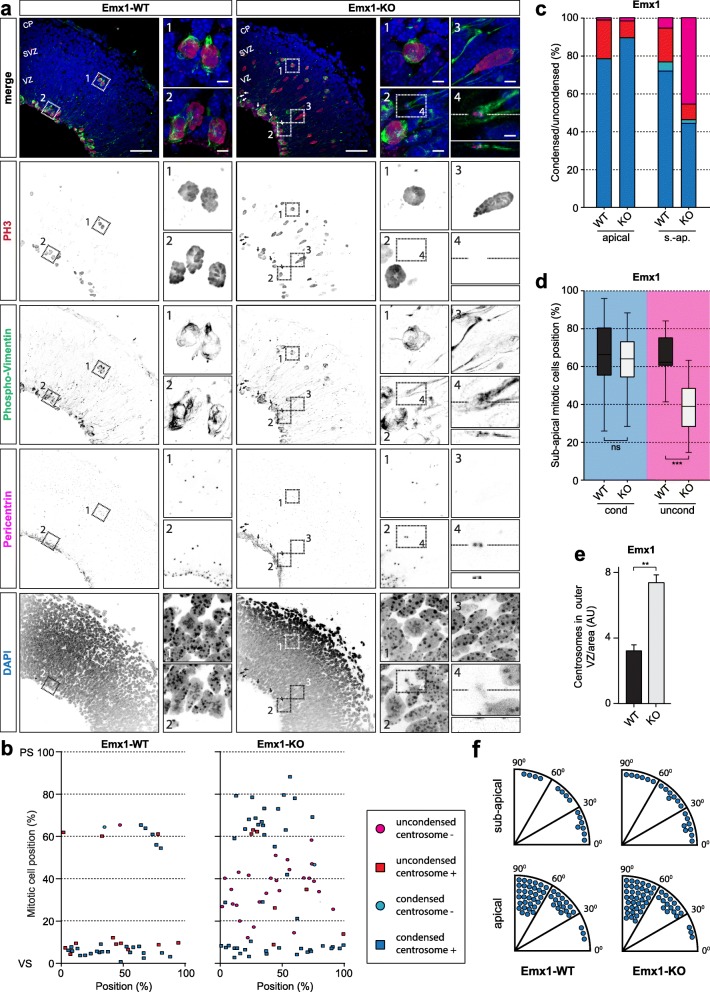
Impaired mitotic progression of a subset of ectopically dividing progenitors is related to positioning of the nucleus and centrosomes. **a**. Coronal cryo-sections of E14.5 cortices from *Bicd2*^*fl/fl*^*;Emx1-Cre*^*−/−*^ (=Emx1-WT) and *Bicd2*^*fl/fl*^*;Emx1-Cre*^*−/+*^ (=Emx1-KO) mice. Cortical sections were stained with the DNA marker DAPI (blue), the centrosome marker Pericentrin (magenta) and the mitotic markers Phospho-Vimentin (green) and Phospho-Histone H3 (PH3, red). Arrows showing apical process of dividing cells containing centrosomes away from the ventricular surface. Zooms showing location of centrosomes (Pericentrin) and chromatin condensation (DAPI) in apical and sub-apical mitotic cells. Emx1-WT + Emx1-KO: Zoom 1: condensed subapical dividing cells with centrosomes. Zoom 2: apical mitotic cells with condensed chromatin and with centrosomes. Emx1-KO: Zoom 3: sub-apical mitotic cell with uncondensed chromatin and without centrosomes. Zoom 4: detached apical process containing centrosomes of mitotic cell in EMX-KO Zoom 3. Scale bars are 50 μm for overview, 5 μm for panels 1–3, and 2 μm for panel 4. **b.** Distribution of mitotic cells with condensed or non-condensed chromatin, and with or without perinuclear centrosomes, as relative position over the cortical longitude from VS to PS (in %). **c.** Quantification of chromatin condensation and perinuclear centrosomes in apical and sub-apical mitotic cells (in %) (*N* = 5–6, *n* = 15–50). **d.** Relative position of sub-apical mitotic cells with condensed or non-condensed chromatin, over the cortical longitude from VS to PS (in %) (*N* = 5–6, *n* = 15–50). **e.** Number of centrosomes per area in the outer VZ (*N* = 6–7, *n* = 10–80). **f.** Cleavage plane orientation relative to the VS based on Pericentrin and DAPI staining in apical and sub-apical mitotic cells. CP: cortical plate, PS: pial surface, SVZ: subventricular zone, VS: ventricular surface, VZ: ventricular zone. s.-ap: sub-apical. *** *p* < 0.001, ** *p* < 0.005, ns = not significant; error bars are ±SEM. Used tests: Kruskal Wallis test with Dunn’s multiple comparisons (**d**), unpaired t-test (**e**)

To examine how the abnormal positions of PH3+ nuclei are related to cell-cycle progression and the apical-basal organization of dividing RGPs, we co-stained for Pericentrin, marking the centrosome, and Phospho-Vimentin (PVim), marking intermediate filaments of mitotic RGPs. Co-localization of centrosomes and PVim was used to determine the location of centrosomes within the mitotic cell. Additionally, to investigate whether the ectopic dividing cells are impaired in the progression through mitosis, the proportion of nuclei with condensed chromatin was determined using DAPI staining (Fig. 7a**, zooms**). In the control cortices most PH3+ nuclei at the VS and in the SVZ had condensed chromatin and centrosomes in the adjacent cytoplasm (Fig. 7c). In Emx1-KO cortices however, a large proportion of ectopic sub-apical dividing progenitors showed uncondensed chromatin and centrosomes were not located proximal to the chromatin, while the sub-apical dividing progenitors with condensed chromatin retained centrosomes in the adjacent cytoplasm. Since centrosomes are normally present at the VS in the apical process of RGPs, these results led us to hypothesize that the condensed nuclei with centrosomes are detached from the VS. Cells in the VZ lacking an apical attachment move into the SVZ, so detached cells are expected to be located further from the VS [50]. Therefore, the distance from sub-apical PH3+ nuclei to the VS was determined for condensed and uncondensed nuclei. In Emx1-WT, condensed and non-condensed sub-apical PH3+ nuclei were located at distances corresponding to the SVZ (Fig. 7d). In Emx1-KO the distance to the VS was significantly shorter, corresponding to the upper VZ for uncondensed but not for condensed PH3+ nuclei. These results are consistent with progenitors with condensed nuclei being released from the VS. Although PVim stained only some of the radial processes of mitotic cells, it showed that the sub-apical progenitors with uncondensed, but not those with condensed nuclei, had a radial morphology characteristic of RGPs, and at least a portion of the uncondensed cells retained an apical process (Fig. 7a). We observed centrosomes located in these apical processes of mitotic sub-apical progenitors which were located at greater distances from the VS than would be expected for centrosomal migration towards the nucleus in late G-2 [60] (Fig. 7a**, arrows + zoom 4**). In addition, the number of centrosomes per area is increased in the upper VZ (7.38 ± 0.71 in Emx1-KO compared to 3.24 ± 0.36 in control littermates; Fig. 7e), suggesting that centrosomes release from the VS in these RGPs. A tilted cleavage plane in RGPs is able to cause detachment from the VS [50], however no difference in cleavage plane orientation was observed between Emx1-WT and Emx1-KO (Fig. 7f). In summary, loss of BICD2 in RGPs causes impaired mitotic progression in progenitors in the upper VZ with a radial morphology and lacking adjacent centrosomes, while progenitors in the SVZ with adjacent centrosomes and no radial orientation did not show this impaired mitotic progression.

Although the precise mechanisms of INM and subsequent cell division at the VS are still largely unclear, they are thought to be essential for the proper temporal regulation of progenitor proliferation and differentiation [3, 12, 21]. To investigate the impact of the AP division at ectopic position in Emx1-KO mice on progenitor proliferation and differentiation, we quantified the number of PAX6+ APs and TBR2+ iBPs. We found the number of PAX6+ APs to be slightly increased in Emx1-KO mice (Fig. 6j), while the number of TBR2+ iBPs was in trend but not significantly decreased (Fig. 6k). This suggests that the ectopic cell division of APs after BICD2 depletion has a moderate impact on the number of APs, but not on the number of iBP cells. Hence, these data suggest that the reduced cortical diameter and disrupted cortical lamination in both Nex-KO and Emx1-KO is not primarily caused by defects in neurogenesis, but rather by the loss in post-mitotic neurons.

## Discussion

In this study, we show that the cell-intrinsic expression and function of BICD2 in excitatory cortical neurons is essential for proper radial neuron migration and neocortical development in vivo. We generated two cell-type specific conditional *Bicd2* KO mouse lines and compared the corticogenesis in the Emx1-KO with the neuron-specific Nex-KO. In contrast to the expression and function of BICD2 in the cerebellum, and to the downregulation of *Bicd2* by RNAi via IUE, our results indicate that the development of the mouse neocortex in vivo mainly depends on *Bicd2* expression and function in post-mitotic neurons, rather than in RGPs.

Loss of BICD2 in cortical neurons disturbs corticogenesis by impeding the radial migration of upper-layer excitatory neurons and formation of the classical mammalian inside-out cortex and interferes with the formation of well-bundled axon tracts in the IZ. Interestingly, early-born neurons which have to migrate shorter distances were much less affected in their migration in BICD2 depleted mice than late-born, far travelling neurons. It is widely accepted that early-born neurons show different radial migratory behavior than late-born neurons. These subsets of neurons are regulated by distinct cellular mechanisms [28]. In early cortical development, neurons do not pursue a multipolar, locomotion and terminal somal translocation mode. Instead, first-born neurons inherit the long basal process from their RGPs [32]. This process is attached to the pial surface and after detaching from the VZ, the neurons migrate upwards by continuous somal translocation [9, 40]. Later, when the IZ starts to form, later-born neurons will first migrate while they are multipolar until they reach the top of the IZ. There, they become bipolar and change to a locomotion mode which is characterized by a continuous growth of the leading edge and the saltatory movement of the nucleus which follows the leading edge growing in the direction of the pia [34, 46]. When the leading process reaches the pia, the tip anchors to the pia and the nucleus migrates via terminal somal translocation smoothly up the leading process. The leading process appears to function as a `grapple` for towing the soma with the nucleus [9]. Despite the fact that the migration of upper-layer neurons in locomotion mode occurs in a RGP-guided manner [9, 40], and slightly disorganized RGP fibers were observed in Emx1-KO, the migration defects we observed in the Emx1-KO were the same as in the neuron-specific Nex-KO. This indicates that radial migration in the cortex depends on the cell-intrinsic function of BICD2 in neurons and is not caused by non-cell-intrinsic effects via RGPs. This neuronal cause of the observed defects in the cerebral cortex corresponds to the neuronal cause shown in *Drosophila* [30], pointing to a neuronal basis for SMALED2A/B in patients.

We speculate that the long-distance movement of the nucleus in the locomotion mode is regulated by distinct cell-intrinsic molecular mechanisms, which depend much more on dynein and the coupling of the nuclear envelope to dynein via RANBP2 and BICD2 than the short-distance migration of early-born deeper-layer neurons. In line with this, TBR1+ deeper-layer neurons showed no significant impairment in their radial migration in both *Bicd2* cKO lines (Additional file 2: **Fig. S2**), while upper-layer SATB2+ or CUX1+ cells were severely affected in their locomotion (Figs. 1,2). These observed altered distributions in cKO mice suggest defects in radial migration of these neurons and raises the question whether loss of BICD2 leads to a global inversion of cortical layering, or is caused by later born neurons being unable to cross layers of previously generated neurons and thereby failing to reach more superficial destinations. If these neurons fail to reach more superficial destinations, the question remains if this is due to a failure to migrate or a delay in neuronal migration. Further studies will have to elucidate if *Bicd2* cKO mice have a global inversion of cortical layering, or if the observed defects are the result of non-migrating layer II/III neurons, or delayed radial neuronal migration. Interestingly, the upper-layer neurons that have to migrate longer distances through the cortical plate were mainly affected in their migration in cKO mice. The immuno-stained nuclei of upper-layer neurons in Nex-KO and Emx1-KO mice were found in the upper SVZ and IZ at a position in the developing cortex where migrating neurons transition from multipolar to bipolar cell morphology and switch from multipolar migration to bipolar locomotion migration mode. For the transition from multipolar to bipolar the regulation of MT and actin dynamics and the reorganization of the cytoskeleton are known to be essential and many microtubule-regulating factors are involved in the multipolar-to-bipolar transition. Interfering with these processes impairs the required morphological changes of the migration neurons before entering the CP, resulting in an accumulation of non-migratory multipolar neurons in the SVZ [40]. Therefore, it is plausible that BICD2 is required for this morphological transition. However, the depletion of BICD2 does not appear to impede the this transition, but instead mainly impairs bipolar locomotion in the CP (Fig. 3), pointing to the different molecular regulation mechanisms at distinct steps of cortical migration and the specific role of BICD2 in dynein mediated transport mechanisms during radial migration in the neocortex. For the migration of bipolar neurons in locomotion mode in the CP, long-distance MT-based transport mechanisms of cell organelles become predominant.

Our labeling of individual neurons in *Bicd2* cKO mice via ex vivo electroporation demonstrates the essential role of BICD2 in the nuclear migration of bipolar locomoting neurons. In contrast to the unimpeded cytoskeleton-dynamic based mechanisms like outgrowth and elongation of leading edges and axons, the migration of neuronal soma was severely impaired in BICD2 depleted cortical neurons. This defect could be fully rescued by the overexpression of wildtype BICD2 in Emx1-KO and Nex-KO cortices. In addition to BICD2_FL rescue, we also attempted rescue experiments with mutant BICD2 to address the cell-intrinsic cellular and molecular function of specific BICD2 domains in cortical migration in vivo. While the full-length wildtype BICD2 fully rescued neuronal migration phenotypes, expression of BICD2_S107L, which is the most commonly found *Bicd2* mutation in SMALED2A patients and has been suggested to increase binding to dynein-dynactin only partially rescues the KO phenotype. Similar to BICD2_FL rescue, migrating neurons could reach the upper layers of the cortex, suggesting partially restored migration capabilities. While it seems that overexpression of SMALED2A mutants does not impair locomotion mode migration, we identified the SMALED2B mutation R694C as the only point mutation which could not restore neuronal migration defects in the mouse neocortex. R694C, which has recently been reported to be associated with cortical malformations in patients, was the only tested mutant BICD2 that failed to rescue neuronal migration defects in Nex-KO mice. Since endfeet positions appeared unaffected and just the soma were found at lower position than after the successful rescue with BICD2_FL, it is tempting to speculate that the mutated region in BICD2_R694C is specifically important for nuclear migration in locomoting neurons. R694C is located in the third coiled coil domain of BICD2, which is necessary for binding to RAB6 and RANBP2 [52]. In mitotic cells, it is known that there is a cell-cycle regulated switch between RAB6 and RANPB2 binding [52]. Future research will have to elucidate if such a switch also occurs in post-mitotic neurons, as the interaction between the nuclear envelope and dynein during neuronal migration in locomotion mode is thought to be relevant. Interestingly, the K758M and E774G mutants, which are also located in CC3 and known to have no or reduced RAB6 binding, could partially rescue the observed KO phenotype. Therefore, it seems likely that the R694C mutation has a different impact than these two mutations on the functionality of the CC3 domain.

In addition to impaired somal migration in cortical neurons of the developing cortex, we found that the depletion of BICD2 also caused severe defects in Golgi organization in CP neurons (Additional file 3: **Fig. S3**). Since our single cell labeling with GFP transfection via EVE or by placing DiI crystals clearly revealed that CP neurons in the cKO mice do become bipolar and form a leading edge, we conclude that the observed Golgi disorganization is not a secondary effect of non-polarization and not forming a leading edge in radial orientation, but a specific result of *Bicd2* loss-of-function in bipolar cortical neurons. Therefore it is also possible that the interaction of BICD2 with Golgi-bound RAB6 might be relevant for proper cortical neuron migration, possibly via regulating the dynein-dependent elongation of the trans-Golgi into the leading edges. So far, it has not been determined whether alterations at amino acid 694 affect BICD2 interactions with RAB6 or RANBP2. Future studies will have to dissect these interactions and their relevance in cortical neurons, as the other two mutations in the third coiled coil domain were able to rescue neuronal migration defects. This suggests that BICD2-RAB6 interaction might not be essential to locomotion mode migration, but possibly in neuronal survival. We observed increased cell death after depletion of BICD2, and since the K758M homologue is a lethal mutation in *Drosophila*, the interaction with RAB6 might be important for Golgi integrity and as such cell survival.

With the Golgi-related phenotypes of *Bicd2* cKO mice in mind, it also becomes interesting to look at minor characteristics of cKO migratory neurons which can be visualized using single cell labeling via EVE: we noticed that the endfeet of the leading edges in BICD2 deficient mice which still reached the marginal zone, showed more extensive branching (Fig. 3a,b). Initiation of neurite branching occurs randomly, and MT stabilization contributes to branch maintenance [9]. The organization of the Golgi apparatus and MT organization and stabilization are known to influence each other [18, 29] and MT abnormalities are known to cause Golgi fragmentation [23]. As such, it is tempting to speculate that the observed disorganized Golgi in neurons of cKO mice might be an indication for changes in MT organization. Alternatively, the presence of Golgi fragments in leading edge branches might locally influence the branching process.

Notably, the Golgi disorganization was not a secondary effect of non-polarization or apoptosis, but was a general defect observed in most neurons in the CP of the cKO mice. BICD2 deficient neurons also acquired a bipolar morphology and the majority of neurons with disturbed Golgi organization were still negative for cleaved Caspase-3. We speculate that the increased cell death in developing *Bicd2* cKO cortices might be the result of impaired neuronal migration and Golgi disorganization rather than vice versa. Notably, the severely enhanced apoptosis in the maturing SP and CP neurons of Nex-KO and Emx1-KO does not seem to significantly alter total number of excitatory CP neurons (Figs. 1,2). However, in view of the total amount of neurons in the CP, the relatively small number of cell affected by increased cell death may fail to illicit a significant reduction in this total number.

While cortical malformations seem to be caused by the loss of BICD2 function in post-mitotic neurons, we observed notable mitotic defects in Emx1-KO mice. In agreement with previously reported effects of *Bicd2* knockdown [21], reduced progenitor division at the VS of Emx1-KO mice in vivo was observed: we found that the spatiotemporal regulation of the RGP cell cycle was affected in Emx1-KO mice (Figs. 6,7) and that an increased amount of RGPs failed to undergo INM. Interestingly however, the number of cells undergoing mitosis was not reduced: we found that RGPs still underwent mitosis, but at an ectopic position. This KO-phenotype is a novel finding and appears to be specific for BICD2 function in progenitor proliferation and differentiation*.* The changed morphology, chromatin condensation and location of the nucleus and centrosome of sub-apical PH3+ progenitors in Emx1-KO mice hint at the presence of two distinct progenitor populations. One population of PH3+ progenitors retains a radial morphology but is impaired in mitotic progression, possibly because the nucleus is stuck in the upper VZ and not able to reach the centrosome. In the second population of progenitors, in which the centrosome lies adjacent to the nucleus, mitotic progression is not impaired. This might be caused by a detachment from the VS, which is supported by the more basal position of their nuclei and lack of radial morphology. Since we observe that centrosomes localized within apical processes of sub-apical mitotic cells, it is possible that the centrosome migrates towards the nucleus when apical nuclear migration is impaired. This could cause a subsequent detachment from the VS, since centrioles at the VS are required for apical attachment by forming the basal body of the primary cilium at the apical end feet [5, 22, 60].

In summary, our comparative studies of cell-type specific *Bicd2* conditional knock-out mice show for the first time that dynein-adaptor protein BICD2 has an essential cell-intrinsic role in radial neuronal migration and neuronal survival in the mammalian neocortex. Systematic comparison between Nex- and Emx1-driven knock-out mice allows us to confidently state that neuron-specific function of BICD2, rather than function of BICD2 in neurogenesis, is important for proper cortical development. The fact that we could rescue neuronal migration defects by overexpressing BICD2, but not by overexpressing mutant BICD2_R694C – associated with cortical malformations in humans – in cKO background, might provide an explanation for the cortical defects observed in patients. As such, loss of cell-intrinsic BICD2 functions in radially migrating cortical neurons might explain PMG-like malformations in humans.

## Materials and methods

### Animals

All applicable international, national, and institutional guidelines for the care and use of animals were followed. All experiments with material from mice were performed in compliance with the guidelines for the welfare of experimental animals issued by the Government of The Netherlands, and were approved by the Animal Ethical Review Committee (DEC) of Utrecht University (permit number 2014.I.03.020 and AVD1080020173404).

### Generation of conditional knock-out mice

To generate the conditional BICD2 KO mouse lines *Bicd2*^*fl/fl*^*;Nex-Cre*^*+/−*^ mice and *Bicd2*^*fl/fl*^*;Emx1-Cre*^*+/−*^ homozygous floxed BICD2 mice [24] were first crossed with heterozygous Nex-Cre or Emx1-Cre mice. The *Bicd2*^*fl/+*^*;Nex-Cre*^*+/−*^ *or Bicd2*^*fl/+*^*;Emx1-Cre*^*+/−*^ offspring was crossed or backcrossed with *Bicd2*^*fl/fl*^ mice to establish *Bicd2*^*fl/fl*^*;Nex-Cre*^*+/−*^ and *Bicd2*^*fl/fl*^*;Emx1-Cre*^*+/−*^ cKO mouse lines. For all experiments *Bicd2*^*fl/fl*^*;Nex-Cre*^*+/−*^ or *Bicd2*^*fl/fl*^*;Emx1-Cre*^*+/−*^ mice were backcrossed with *Bicd2*^*fl/fl*^ mice and *Bicd2*^*fl/fl*^*;Nex-Cre*^*+/−*^ (referred to as Nex-KO) and *Bicd2*^*fl/fl*^*;Nex-Cre*^*−/−*^ mice (referred to as Nex-WT) or *Bicd2*^*fl/fl*^*;Emx1-Cre*^*+/−*^ (referred to as Emx1-KO) and *Bicd2*^*fl/fl*^*;Emx1-Cre*^*−/−*^ (referred to as Emx1-WT) from the same litter were analyzed.

### Genotyping of cKO mice

DNA was isolated from earclips (adult mice) or tail tissue (embryos) and standard or touchdown genotyping-PCRs were performed using DreamTaq DNA polymerase (ThermoScientific) and the following primers for detecting the *Bicd2* floxed allele: Primer 75 CGGCGGCATCAGAGCAGCCG; Primer 76 GTAGCACTTCAGGAACATCCATGC; Primer 77 GGAGAAGATCTCATCTTGGCAGG, for detecting the Nex-Cre allele: Primer Nex_as 3132 AGAATGTGGAGTAGGGTGAC; Primer Nex148_s 3131 GAGTCCTGGAATCAGTCTTTTTC; Primer Cre_a 2409 CCGCATAACCAGTGAAACAG, for detecting the Emx1-Cre allele: Primer 1084 GCGGTCTGGCAGTAAAAACTATC; Primer 1085 GTG AAACAGCAT TGCTGTCACTT; Primer 4170 AAGGTGTGGTTCCAGAATCG; Primer 4171 CTCTCCACCAGAAGGCTGAG.

### DNA constructs

BICD2 mutant constructs were generated from wildtype mouse BICD2 (annotated under the accession number AJ250106). Mouse and human BICD2 sequences were aligned and residues corresponding to human mutations were identified. Mutations were introduced using PCR-based strategies (primer list: Additional file 6: Table S1). Constructs were cloned into the pGW1-CMV (British Biotechnology) expression vector. In addition, we used the following plasmids that were previously described: pGW1-GFP-BICD2 [48], pGW1-GFP-BICD2-K758M [49], MARCKS-GFP [47].

All constructs were generated by PCR amplification using primers mentioned above (Additional file 6: Table S1). Additional information available on request.

### Antibodies and reagents

Antibodies used in this study: Mouse anti-Actin (clone nr. C4, MAB1501R, Chemicon); Rabbit anti-BICD2 (HPA023013, Atlas Antibodies); Rabbit anti-BICD2 #2293 (homemade, [24]); Rabbit anti-BICD1/2 #2294 (homemade, [24]); Rabbit anti-Caspase-3 (clone nr. Asp715, 9661S, Lot 43, Cell Signalling); Rat anti-CTIP2 (ab18465, Abcam); Rabbit anti-CUX1 (Santa Cruz); Chicken anti-DCX (anti-doublecortin, ab153668, Abcam), Rabbit anti-GFP (598, MBL; ab290. Abcam); Mouse anti-GM130 (610823, BD), Mouse anti-Nestin (611658, BD), Mouse anti-Nestin (rat 401, MAB535, Millipore); Mouse anti-NeuN (MAB377, Millipore); Rabbit anti-NeuN (ab177487, Abcam); Chicken anti-Neurofilament_heavy_200kD (ab72996, Abcam); Rabbit anti-PAX6 (ab5790, Abcam); Rabbit anti-PAX6 (clone nr. Poly19013, 901301, ITK Diagnostics); Mouse anti-PAX6 (clone nr. AD2.38, ab78545, Abcam); Rabbit anti-Pericentrin (923701, ITK Diagnostics); Mouse anti-Pericentrin (611815, BD), Mouse anti-Phospho-Histone H3 (Ser10) (clone nr. 6G3, 9706, Cell Signaling); Rabbit anti-Phospho-Histone H3 (Ser10); Mouse anti-Satb2 (clone nr. SATBA4B10, ab51502, Abcam); Rabbit anti-TBR1 (ab31940, Abcam); Rabbit anti-TBR2 (ab23345, Abcam); Mouse anti-Tubulin-alpha (clone nr. B-5-1-2, T-5168, Sigma); Mouse IgG2b anti-Vimentin phospho S55 (clone 4A4, ab22651, Abcam); Alexa405-, Alexa488-, Alexa568- Alexa594 and Alexa647-conjugated secondary antibodies (Life Technologies); IRdye800CW-conjugated secondary antibodies (LI-COR Biosciences). Other reagents used in this study include: DAPI (Sigma); Fast Green FCF (F7252, Sigma): NeuroTrace™ DiI Tissue-Labeling Paste (N22880, ThermoFisher).

### Immunoblotting

Whole cortical extracts were made by isolating cortices from E17.5 brains from individual embryos and homogenizing and lysing the cortices in modified RIPA buffer (50 mM HEPES, pH 7.4, 1% Sodium deoxycholate, 20 mM Na_4_P_2_O_7_, 0.1% SDS, 150 mM NaCl, 10% Glycerol, 1.5 mM MgCl_2_ and complete protease inhibitor (Roche)). Extracts were centrifuged and the supernatants were boiled in SDS-page sample buffer containing DTT and 15 mg loaded on a Tris-Glycine SDS-polyacrylamide gel and blotted on nitrocellulose membranes. Blots were blocked in 4% milk in PBS-T (0.05% Tween20) followed by primary and secondary antibody incubation prior scanning with an Odyssey infrared imaging system (Li-COR Biosciences).

### Immunohistochemistry

Brains were isolated at embryonic day 14.5 or 17.5, shortly rinsed with PBS, fixed in paraformaldehyde for 2.5 or 4.5 h at 4 °C respectively, washed with PBS and transferred to 30% sucrose overnight for cryoprotection prior to freezing in Jung Tissue freezing medium (Leica). Brains were cut in 12 μm coronal sections on a freezing microtome (Leica) and collected on Thermo Scientific Superfrost Plus™ microscope slides. Sections were washed in PBS, heated in a microwave for antigen retrieval for 10 min in Sodium Citrate buffer (10 mM, pH 6) at 97^o^ C, washed in PBS and blocked for 1 h using 10% normal goat serum with 0.2% Triton X-100 in PBS followed by primary and secondary antibody incubation in blocking solution, both at 4^o^ C ON. Slides were mounted using Vectashield mounting medium with DAPI (Vectorlabs) and sealed with nail polish prior to confocal microscopy.

### Ex vivo electroporation

Embryonic heads were isolated at E14.5, and brains were electroporated with 1.5µl DNA mixture containing MARCKS-GFP vector and the indicated BICD2 constructs dissolved in Milli-Q water with 0.05% Fast Green FCF dye (Sigma). DNA mixture was injected in the lateral ventricles of the embryonic brains using borosilicate glass micro-pipettes (World Precision Instruments) and a PLI-100A Pico-liter injector (Warner Instruments). Brains were electroporated with platinum plated tweezer-electrodes (Nepagene) using an ECM 830 Electro-Square-Porator (Harvard Apparatus) set to three unipolar pulses at 30 V (100 ms interval and pulse length). Embryonic brains were then isolated and collected in ice-cold cHBSS, embedded in 3% SeaPlaque GTG Agarose (Lonza) in cHBSS and sectioned coronally in 300 µm slices using a VT1000 S Vibratome (Leica). Slices were collected on poly-L-lysine and laminin-coated culture membrane inserts (Falcon), placed on top of slice culture medium (70% v/v Basal Medium Eagle, 26% v/v cHBSS, 20 mM D-Glucose, 1 mM L-Glutamine, 0.1 mg/mL penicillin/streptomycin) and cultured for 4 days prior to fixation with 4% paraformaldehyde in PBS. Slices were then blocked and permeabilized in 10% Normal Goat Serum with 0.2% Triton X-100 in PBS followed by primary (anti-GFP) and secondary antibody incubation (containing DAPI) in blocking solution. Slides were mounted using Vectashield mounting medium with DAPI (Vectorlabs) and sealed with nail polish prior to confocal microscopy.

### DiI labeling

At embryonic day 17.5 brains were isolated, shortly rinsed with PBS, fixed in paraformaldehyde for 1 h at 4 °C, washed with PBS and embedded in low melting agarose. Brains were cut in 200 μm coronal sections on a vibratome (Leica), collected on frosted microscope slides and covered with PBS. Gel containing DiI crystals (NeuroTrace™ DiI Tissue-Labeling Paste, N22880, ThermoFisher) was placed with a needle in the IZ. Sections were incubated for 30 h at 37 °C in a wet chamber to label the membranes of the DiI-targeted contra-lateral and cortico-fugal projecting neurons in the developing cortex. Z-stack acquisitions were taken within 4 h after incubation using conventional laser confocal microscopy.

### Immunohistochemistry microscopy

Immunohistochemistry microscopy of cryosections: Confocal laser scanning microscopy was performed using a LSM-700 system (Zeiss) with a Plan-Apochromat 20x NA 0.8, an EC Plan-Neofluar 40x NA1.30 Oil DIC Plan-Apochromat, or a Plan-Apochromat 63x NA 1.40 oil DIC. Z-stacks were selected to cover the entire section and taken in software (ZEN) suggested optimal size steps.

Immunohistochemistry microscopy of fixed organotypical slice cultures after ex vivo electroporation: Confocal laser scanning microscopy was performed using a LSM-700 system (Zeiss) with a Plan-Apochromat 20x NA 0.8 objective or using a SP8 system (Leica) with a HCX PL FLUOTAR L 20x objective. Z-stacks were selected to cover the entire section and taken in software (ZEN) suggested optimal size steps.

### Image analysis and quantification

Analysis and linear image processing was performed in FIJI. All quantifications (unless differently indicated) were performed using the same microscope settings across experiments.

#### Cell positioning

Numbers of, and positions of different kinds of cells (e.g. TBR2+, NeuN+ or mitotic cells) were determined using the FIJI cell counter plug-in. Cell positions were transformed from absolute locations to relative positions on an x and y-axis where the y-axis goes from 0% (ventricular surface) to 100% (pial surface), and the x-axis goes from 0 to 100% in width (specific crops, figure specific; see figure legends).

#### Neurofilament band

Location and width of neurofilament band was determined using background substracted values obtained by drawing a linescan across the cortex (VS - > PS). A specific threshold was applied on relative immunofluorescent signal intensity to determine the start and end of the NF band.

#### Ex vivo electroporation

Positions of migrating neuronal soma and corresponding endfeet were determined using the FIJI cell counter plug-in. All GFP+ cells located above 30% relative position (on the y-, VS-PS, axis) were counted as migrating and included in this analysis. Cell positions were transformed from absolute locations to relative positions on an x and y-axis where the y-axis goes from 0% (ventricular surface) to 100% (pial surface), and the x-axis goes from 0 to 100% in width (specific crops; see figure legend). Length of the leading edge was determined by substracting relative somatic postion from relative endfeet position.

#### Mitotic progression

The condensation state of chromatin was allocated to counted cells using DAPI staining and the presence of perinuclear centrosomes was determined by Pericentrin staining within the phospho-vimentin positive area surrounding the nucleus. Mitotic cells were counted as apical when the nucleus was within 30 μm of the Ventricular surface and as sub-apical otherwise. Centrosomes were counted using cell-counter in the centrosome poor regions in the outer ventricular zone. Cleavage plane orientation was determined using the angle tool and measuring the angle between a line connecting the two centrosomes and a line in the radial direction of the cortex.

### Statistical analysis

All statistical details of experiments, including the definitions and exact values of *N* & *n*, and statistical tests performed, can be found in figure legends. All data was checked for normality by Shapiro–Wilk test. We define the number of mice as *N* (e.g. three mice used for Nex-WT and four mice used for Nex-KO would be given as *N* = 3–4), and the number of counted cells/individual data points as *n* (e.g. sample with lowest numbers of positive cells has 200 cells, sample with highest number has 300 cells would be given as *n* = 200–300). For each genotype, mice come from at least two different litters. Data processing and statistical analysis were done in Excel and GraphPad Prism 7. Significance was defined as: ns = not significant or *p* > 0.05, * for *p* < 0.05, ** for *p* < 0.005, *** for *p* < 0.001. Error bars are ±SEM.

## Supplementary information

**Additional file 1: Fig. S1.** *Bicd2* is expressed by RGPs and post-mitotic neurons in the cortex of control mice, but abolished after cell-type-specific depletion of BICD2 in cKO mice. **a.** Coronal cryo-sections of E17.5 brains from cell-type-specific conditional *Bicd2* KO and control mice - *Bicd2*^*fl/fl*^*;Nex-Cre*^*+/−*^ (=Nex-KO), *Bicd2*^*fl/fl*^*;Emx1-Cre*^*+/−*^ (=Emx1-KO) and *Bicd2*^*fl/fl*^*;Cre*^*−/−*^ (=Nex-WT/Emx1-WT) respectively - were stained against BICD2. Scale bars are 500 μm. **b.** Coronal cryo-sections of E17.5 cortices from Nex-KO, Emx1-KO and Nex-WT/Emx1-WT mice were stained against BICD2 (red). DAPI is shown in blue. Scale bars are 100 μm. **c.** Zooms from BICD2 staining shown in (**b**). **d + e.** Western Blots (WBs) of whole E17.5 cortex lysates from Nex-KO, Emx1-KO, and their control littermates Nex-WT and Emx1-WT. WBs were stained with commercial anti-BICD2 (atlas), home-made anti- BICD2 (#2294), home-made anti-BICD1/2(#2293), anti-actin or anti-tubulin. M = marker. Molecular weight of the marker bands are indicated on the left in kDa. (*N* = 3). **f.** Radial diameter of the cortex from ventricular to pial surface of Nex-WT, Nex-KO, Emx1-WT and Emx1-KO mice (in μm) (*N* = 16–17). *** *p* < 0.001, ns = not significant; error bars are ±SEM.CP: cortical plate, SVZ: subventricular zone, VZ: ventricular zone. Used test: Kruskal Wallis test with Dunn’s multiple comparisons.

**Additional file 2: Fig. S2.** Cell-type specific depletion of BICD2 does not affect migration of first-born CP neurons but impairs the axonal organization of contra-lateral and cortico-fugal projecting neurons in the IZ. **a.** Coronal cryo-sections of E17.5 cortices from cell-type-specific conditional *Bicd2* KO mice and their control littermates - *Bicd2*^*fl/fl*^*;Nex-Cre*^*+/−*^ (=Nex-KO), *Bicd2*^*fl/fl*^*;Emx1-Cre*^*+/−*^ (=Emx1-KO), *Bicd2*^*fl/fl*^*;Nex-Cre*^*−/−*^ (=Nex-WT) and *Bicd2*^*fl/fl*^*;Emx1-Cre*^*−/−*^ (=Emx1-WT) respectively - were stained against Neurofilament heavy chain (NF) (red) and the cortical layer VI marker TBR1 (green). Scale bars are 100 μm. **b.** Quantification of the relative frequency of NF+ axons over the cortical longitude from ventricular (VS) to pial surface (PS) (%, binned in centers) and their gaussian distribution and the average NF intensity (AU) for Nex-WT and Nex-KO mice (left) and Emx1-WT and Emx1-KO mice (right). **c**. Width of the band with NF+ axons in μm (*N* = 4). **d + e.** Selected area from VS to PS and 156.3 μm width (left panels) and graphical representation of the relative position of TBR1+ cells over the cortical longitude from VS to PS (in %) and 156.3 μm in width (in %) (right panels) for Nex-WT and Nex-KO mice (**d**) and Emx1-WT and Emx1-KO mice (**e**). Scale bars in left panels are 50 μm. **f.** Relative position of TBR1+ layer VI neurons in the CP and NF+ axons in the IZ over the cortical longitude from VS to PS (in %). Bar represents average top, middle and bottom of the TBR1+ band (*N* = 3, *n* = 122–204). **g.** Number (10^− 3^) of TBR1+ cells per μm^2^ (*N* = 3, *n* = 122–204). **h.** Coronal cryo-sections of E17.5 cortices from Nex-KO, Emx1-KO, Nex-WT and Emx1-WT were stained against Nestin (green) to mark the fibers in RGP processes and Pericentrin (red) to mark the centrosomes. Scale bars are 100 μm. CP: cortical plate, IZ: intermediate zone, PS: pial surface, SVZ: subventricular zone, VS: ventricular surface, VZ: ventricular zone. *** *p* < 0.001, ** *p* < 0.005, ns = not significant; error bars are ±SEM. Used tests: One Way ANOVA with Tukey’s multiple comparisons (**c**), Kruskal Wallis test with Dunn’s multiple comparisons (**f, g**).

**Additional file 3: Fig. S3.** BICD2 is essential for proper Golgi organization in CP neurons. **a + b.** Coronal cryo-sections of E17.5 cortices from *Bicd2*^*fl/fl*^*;Nex-Cre*^*−/−*^ (=Nex-WT) and *Bicd2*^*fl/fl*^*;Nex-Cre*^*+/−*^ (=Nex-KO) mice (**a**) and *Bicd2*^*fl/fl*^*;Emx1-Cre*^*−/−*^ (=Emx1-WT) and *Bicd2*^*fl/fl*^*;Emx1-Cre*^*+/−*^ (=Emx1-KO) mice (**b**) were stained against the trans-Golgi marker GM130 (red) and Neurofilament heavy chain (green) to indicate the IZ. DAPI is shown in blue. For each genotype confocal images in 20x (left) and 40x (right) are shown; scale bars are 100 μm and 50 μm, respectively. **c + d.** 63x zoom confocal images with GM130 stained trans-Golgi in the CP. Scale bars are 10 μm (**c**) and 50 μm (**d**). CP: cortical plate, IZ: intermediate zone, SVZ: subventricular zone.

**Additional file 4: Fig. S4.** DiI labeling of single neurons. Lightly fixed coronal brain sections from E17.5 Nex-WT and Nex-KO mice. DiI crystals were placed in the IZ to label and visualize individual neurons. CP: cortical plate, IZ: intermediate zone, SVZ: subventricular zone.

**Additional file 5: Fig. S5.** Depletion of BICD2 in cortical cells results in an increase of apoptotic cell death in progenitor cell layers at E14.5. **a.** Coronal cryo-sections of E14.5 cortices from cell-type-specific conditional *Bicd2* KO mice *Emx1-Cre*^*+/−*^ (=Emx1-KO) and their control littermates *Bicd2*^*fl/fl*^*;Emx1-Cre*^*−/−*^ were stained against apoptotic marker Caspase-3 (Cas3) (red) and Doublecortin (DCX) as early neuronal marker (green). DAPI is shown in blue. Scale bars are 100 μm. **b.** Zoom of Caspase-3 staining shown in (**a**). Scale bars are 50 μm. **c.** Graphical representation of the relative position of Cas3+ cells over the cortical longitude from ventricular (VS) to pial surface (PS) (both in %). **d.** Number (10^− 3^) of Cas3+ cells per μm^2^ (*N* = 6–12, *n* = 0–21). **e.** Cas3+ cell distribution as relative position over the cortical longitude from VS to PS (in %) for Emx1-WT and Emx1-KO mice. Red circles are individual Cas3+ cell locations of representative samples (*N* = 6–12, *n* = 0–21). *** *p* < 0.001; error bars are ±SEM. used test: unpaired t-test (**d**), Mann Whitney *U* test (**e**).

**Additional file 6: Table S1.** Cloning and sequencing primers.

## Data Availability

The datasets generated during and/or analyzed during the current study are available from the corresponding author on reasonable request.

## References

[1] Alcamo EA, Chirivella L, Dautzenberg M, Dobreva G, Farinas I, Grosschedl R, McConnell SK (2008). Satb2 regulates callosal projection neuron identity in the developing cerebral cortex. Neuron.

[2] Arlotta P, Molyneaux BJ, Chen J, Inoue J, Kominami R, Macklis JD (2005). Neuronal subtype-specific genes that control corticospinal motor neuron development in vivo. Neuron.

[3] Baffet AD, Hu DJ, Vallee RB (2015). Cdk1 activates pre-mitotic nuclear envelope dynein recruitment and apical nuclear migration in neural stem cells. Dev Cell.

[4] Britanova O, de Juan RC, Cheung A, Kwan KY, Schwark M, Gyorgy A, Vogel T, Akopov S, Mitkovski M, Agoston D, Sestan N, Molnar Z, Tarabykin V (2008). Satb2 is a postmitotic determinant for upper-layer neuron specification in the neocortex. Neuron.

[5] Buchman JJ, Tseng HC, Zhou Y, Frank CL, Xie Z, Tsai LH (2010). Cdk5rap2 interacts with pericentrin to maintain the neural progenitor pool in the developing neocortex. Neuron.

[6] Bullock SL, Ish-Horowicz D (2001). Conserved signals and machinery for RNA transport in Drosophila oogenesis and embryogenesis. Nature.

[7] Bullock SL, Nicol A, Gross SP, Zicha D (2006). Guidance of bidirectional motor complexes by mRNA cargoes through control of dynein number and activity. Curr Biol.

[8] Claussen M, Suter B (2005). BicD-dependent localization processes: from Drosophilia development to human cell biology. Ann Anat.

[9] Cooper JA (2013). Cell biology in neuroscience: mechanisms of cell migration in the nervous system. J Cell Biol.

[10] Englund C, Fink A, Lau C, Pham D, Daza RA, Bulfone A, Kowalczyk T, Hevner RF (2005). Pax6, Tbr2, and Tbr1 are expressed sequentially by radial glia, intermediate progenitor cells, and postmitotic neurons in developing neocortex. J Neurosci.

[11] Fiorillo C, Moro F, Yi J, Weil S, Brisca G, Astrea G, Severino M, Romano A, Battini R, Rossi A, Minetti C, Bruno C, Santorelli FM, Vallee R (2014). Novel dynein DYNC1H1 neck and motor domain mutations link distal spinal muscular atrophy and abnormal cortical development. Hum Mutat.

[12] Fousse J, Gautier E, Patti D, Dehay C (2019). Developmental changes in interkinetic nuclear migration dynamics with respect to cell-cycle progression in the mouse cerebral cortex ventricular zone. J Comp Neurol.

[13] Goebbels S, Bormuth I, Bode U, Hermanson O, Schwab MH, Nave KA (2006). Genetic targeting of principal neurons in neocortex and hippocampus of NEX-Cre mice. Genesis.

[14] Goncalves JC, Dantas TJ, Vallee RB (2019). Distinct roles for dynein light intermediate chains in neurogenesis, migration, and terminal somal translocation. J Cell Biol.

[15] Gorski JA, Talley T, Qiu M, Puelles L, Rubenstein JL, Jones KR (2002) Cortical excitatory neurons and glia, but not GABAergic neurons, are produced in the Emx1-expressing lineage. J Neurosci 22:6309-6314. Doi:20026564 10.1523/JNEUROSCI.22-15-06309.2002 PMC6758181 12151506

[16] Grigoriev I, Splinter D, Keijzer N, Wulf PS, Demmers J, Ohtsuka T, Modesti M, Maly IV, Grosveld F, Hoogenraad CC, Akhmanova A (2007). Rab6 regulates transport and targeting of exocytotic carriers. Dev Cell.

[17] Guarnieri FC, de Chevigny A, Falace A, Cardoso C (2018). Disorders of neurogenesis and cortical development. Dialogues Clin Neurosci.

[18] Gurel PS, Hatch AL, Higgs HN (2014). Connecting the cytoskeleton to the endoplasmic reticulum and Golgi. Curr Biol.

[19] Hoogenraad CC, Akhmanova A (2016). Bicaudal D family of motor adaptors: linking dynein motility to cargo binding. Trends Cell Biol.

[20] Hoogenraad CC, Akhmanova A, Howell SA, Dortland BR, De Zeeuw CI, Willemsen R, Visser P, Grosveld F, Galjart N (2001). Mammalian Golgi-associated Bicaudal-D2 functions in the dynein-dynactin pathway by interacting with these complexes. EMBO J.

[21] Hu DJ, Baffet AD, Nayak T, Akhmanova A, Doye V, Vallee RB (2013). Dynein recruitment to nuclear pores activates apical nuclear migration and mitotic entry in brain progenitor cells. Cell.

[22] Insolera R, Bazzi H, Shao W, Anderson KV, Shi SH (2014). Cortical neurogenesis in the absence of centrioles. Nat Neurosci.

[23] Jaarsma D, Hoogenraad CC (2015). Cytoplasmic dynein and its regulatory proteins in Golgi pathology in nervous system disorders. Front Neurosci.

[24] Jaarsma D, van den Berg R, Wulf PS, van Erp S, Keijzer N, Schlager MA, de Graaff E, De Zeeuw CI, Pasterkamp RJ, Akhmanova A, Hoogenraad CC (2014). A role for Bicaudal-D2 in radial cerebellar granule cell migration. Nat Commun.

[25] Kato M (2015). Genotype-phenotype correlation in neuronal migration disorders and cortical dysplasias. Front Neurosci.

[26] Kawauchi T (2015). Cellullar insights into cerebral cortical development: focusing on the locomotion mode of neuronal migration. Front Cell Neurosci.

[27] Kriegstein AR, Noctor SC (2004). Patterns of neuronal migration in the embryonic cortex. Trends Neurosci.

[28] Marin O, Valiente M, Ge X, Tsai LH (2010). Guiding neuronal cell migrations. Cold Spring Harb Perspect Biol.

[29] Martin M, Akhmanova A (2018). Coming into focus: mechanisms of microtubule minus-end organization. Trends Cell Biol.

[30] Martinez Carrera LA, Gabriel E, Donohoe CD, Holker I, Mariappan A, Storbeck M, Uhlirova M, Gopalakrishnan J, Wirth B (2018). Novel insights into SMALED2: BICD2 mutations increase microtubule stability and cause defects in axonal and NMJ development. Hum Mol Genet.

[31] Martinez-Carrera LA, Wirth B (2015). Dominant spinal muscular atrophy is caused by mutations in BICD2, an important golgin protein. Front Neurosci.

[32] Miyata T, Kawaguchi A, Okano H, Ogawa M (2001). Asymmetric inheritance of radial glial fibers by cortical neurons. Neuron.

[33] Molyneaux BJ, Arlotta P, Menezes JR, Macklis JD (2007). Neuronal subtype specification in the cerebral cortex. Nat Rev Neurosci.

[34] Nadarajah B, Brunstrom JE, Grutzendler J, Wong RO, Pearlman AL (2001). Two modes of radial migration in early development of the cerebral cortex. Nat Neurosci.

[35] Neveling K, Martinez-Carrera LA, Holker I, Heister A, Verrips A, Hosseini-Barkooie SM, Gilissen C, Vermeer S, Pennings M, Meijer R, te Riele M, Frijns CJ, Suchowersky O, MacLaren L, Rudnik-Schoneborn S, Sinke RJ, Zerres K, Lowry RB, Lemmink HH, Garbes L, Veltman JA, Schelhaas HJ, Scheffer H, Wirth B (2013). Mutations in BICD2, which encodes a golgin and important motor adaptor, cause congenital autosomal-dominant spinal muscular atrophy. Am J Hum Genet.

[36] Nieto M, Monuki ES, Tang H, Imitola J, Haubst N, Khoury SJ, Cunningham J, Gotz M, Walsh CA (2004). Expression of Cux-1 and Cux-2 in the subventricular zone and upper layers II-IV of the cerebral cortex. J Comp Neurol.

[37] Nishimura YV, Shikanai M, Hoshino M, Ohshima T, Nabeshima Y, Mizutani K, Nagata K, Nakajima K, Kawauchi T (2014). Cdk5 and its substrates, dcx and p27kip1, regulate cytoplasmic dilation formation and nuclear elongation in migrating neurons. Development.

[38] Noctor SC, Martinez-Cerdeno V, Ivic L, Kriegstein AR (2004). Cortical neurons arise in symmetric and asymmetric division zones and migrate through specific phases. Nat Neurosci.

[39] Oates Emily C., Rossor Alexander M., Hafezparast Majid, Gonzalez Michael, Speziani Fiorella, MacArthur Daniel G., Lek Monkol, Cottenie Ellen, Scoto Mariacristina, Foley A. Reghan, Hurles Matthew, Houlden Henry, Greensmith Linda, Auer-Grumbach Michaela, Pieber Thomas R., Strom Tim M., Schule Rebecca, Herrmann David N., Sowden Janet E., Acsadi Gyula, Menezes Manoj P., Clarke Nigel F., Züchner Stephan, Muntoni Francesco, North Kathryn N., Reilly Mary M. (2013). Mutations in BICD2 Cause Dominant Congenital Spinal Muscular Atrophy and Hereditary Spastic Paraplegia. The American Journal of Human Genetics.

[40] Ohtaka-Maruyama C, Okado H (2015). Molecular pathways underlying projection neuron production and migration during cerebral cortical development. Front Neurosci.

[41] Peeters K, Litvinenko I, Asselbergh B, Almeida-Souza L, Chamova T, Geuens T, Ydens E, Zimon M, Irobi J, De Vriendt E, De Winter V, Ooms T, Timmerman V, Tournev I, Jordanova A (2013). Molecular defects in the motor adaptor BICD2 cause proximal spinal muscular atrophy with autosomal-dominant inheritance. Am J Hum Genet.

[42] Rakic P (1972). Mode of cell migration to the superficial layers of fetal monkey neocortex. J Comp Neurol.

[43] Ravenscroft G, Di Donato N, Hahn G, Davis MR, Craven PD, Poke G, Neas KR, Neuhann TM, Dobyns WB, Laing NG (2016). Recurrent de novo BICD2 mutation associated with arthrogryposis multiplex congenita and bilateral perisylvian polymicrogyria. Neuromuscul Disord.

[44] Reck-Peterson SL, Redwine WB, Vale RD, Carter AP (2018). The cytoplasmic dynein transport machinery and its many cargoes. Nat Rev Mol Cell Biol.

[45] Romero DM, Bahi-Buisson N, Francis F (2018). Genetics and mechanisms leading to human cortical malformations. Semin Cell Dev Biol.

[46] Sakakibara A, Sato T, Ando R, Noguchi N, Masaoka M, Miyata T (2014). Dynamics of centrosome translocation and microtubule organization in neocortical neurons during distinct modes of polarization. Cereb Cortex.

[47] Schatzle P, Kapitein LC, Hoogenraad CC (2016). Live imaging of microtubule dynamics in organotypic hippocampal slice cultures. Methods Cell Biol.

[48] Schlager MA, Kapitein LC, Grigoriev I, Burzynski GM, Wulf PS, Keijzer N, de Graaff E, Fukuda M, Shepherd IT, Akhmanova A, Hoogenraad CC (2010). Pericentrosomal targeting of Rab6 secretory vesicles by Bicaudal-D-related protein 1 (BICDR-1) regulates neuritogenesis. EMBO J.

[49] Schlager MA, Serra-Marques A, Grigoriev I, Gumy LF, Esteves da Silva M, Wulf PS, Akhmanova A, Hoogenraad CC (2014). Bicaudal d family adaptor proteins control the velocity of dynein-based movements. Cell Rep.

[50] Shitamukai A, Konno D, Matsuzaki F (2011). Oblique radial glial divisions in the developing mouse neocortex induce self-renewing progenitors outside the germinal zone that resemble primate outer subventricular zone progenitors. J Neurosci.

[51] Splinter D, Razafsky DS, Schlager MA, Serra-Marques A, Grigoriev I, Demmers J, Keijzer N, Jiang K, Poser I, Hyman AA, Hoogenraad CC, King SJ, Akhmanova A (2012). BICD2, dynactin, and LIS1 cooperate in regulating dynein recruitment to cellular structures. Mol Biol Cell.

[52] Splinter D, Tanenbaum ME, Lindqvist A, Jaarsma D, Flotho A, Yu KL, Grigoriev I, Engelsma D, Haasdijk ED, Keijzer N, Demmers J, Fornerod M, Melchior F, Hoogenraad CC, Medema RH, Akhmanova A (2010). Bicaudal D2, dynein, and kinesin-1 associate with nuclear pore complexes and regulate centrosome and nuclear positioning during mitotic entry. PLoS Biol.

[53] Storbeck M, Horsberg Eriksen B, Unger A, Holker I, Aukrust I, Martinez-Carrera LA, Linke WA, Ferbert A, Heller R, Vorgerd M, Houge G, Wirth B (2017). Phenotypic extremes of BICD2-opathies: from lethal, congenital muscular atrophy with arthrogryposis to asymptomatic with subclinical features. Eur J Hum Genet.

[54] Stouffer MA, Golden JA, Francis F (2016). Neuronal migration disorders: focus on the cytoskeleton and epilepsy. Neurobiol Dis.

[55] Stuurman N, Haner M, Sasse B, Hubner W, Suter B, Aebi U (1999). Interactions between coiled-coil proteins: Drosophila Lamin Dm0 binds to the bicaudal-D protein. Eur J Cell Biol.

[56] Swan A, Nguyen T, Suter B (1999). Drosophila Lissencephaly-1 functions with Bic-D and dynein in oocyte determination and nuclear positioning. Nat Cell Biol.

[57] Tabata H, Nakajima K (2003). Multipolar migration: the third mode of radial neuronal migration in the developing cerebral cortex. J Neurosci.

[58] Taverna E, Mora-Bermudez F, Strzyz PJ, Florio M, Icha J, Haffner C, Norden C, Wilsch-Brauninger M, Huttner WB (2016). Non-canonical features of the Golgi apparatus in bipolar epithelial neural stem cells. Sci Rep.

[59] Tsai JW, Bremner KH, Vallee RB (2007). Dual subcellular roles for LIS1 and dynein in radial neuronal migration in live brain tissue. Nat Neurosci.

[60] Tsai JW, Lian WN, Kemal S, Kriegstein AR, Vallee RB (2010). Kinesin 3 and cytoplasmic dynein mediate interkinetic nuclear migration in neural stem cells. Nat Neurosci.

[61] Urnavicius L, Zhang K, Diamant AG, Motz C, Schlager MA, Yu M, Patel NA, Robinson CV, Carter AP (2015). The structure of the dynactin complex and its interaction with dynein. Science.

