# Supplementary figures and images for: Dynein activating adaptor BICD2 controls radial migration of upper-layer cortical neurons in vivo
Source: Acta Neuropathol Commun. 2019 Oct 26;7:162. doi: 10.1186/s40478-019-0827-y (PMC6815425; doi:10.1186/s40478-019-0827-y)

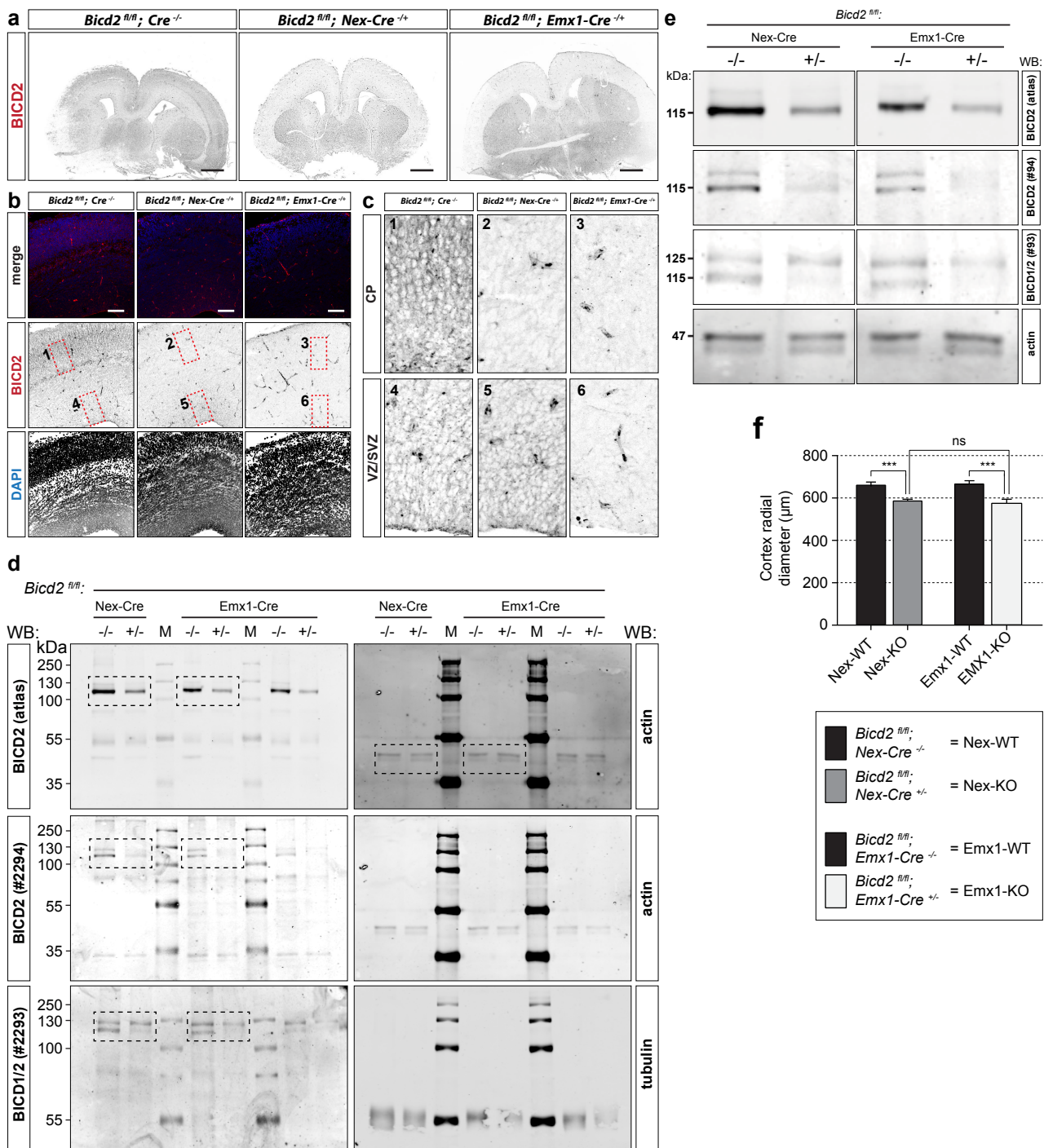

Supplement: Supplementary file 1 — Additional file 1: Fig. S1. Bicd2 is expressed by RGPs and post-mitotic neurons in the cortex of control mice, but abolished after cell-type-specific depletion of BICD2 in cKO mice. a. Coronal cryo-sections of E17.5 brains from cell-type-specific conditional Bicd2 KO and control mice - Bicd2fl/fl;Nex-Cre+/− (=Nex-KO), Bicd2fl/fl;Emx1-Cre+/− (=Emx1-KO) and Bicd2fl/fl;Cre−/− (=Nex-WT/Emx1-WT) respectively - were stained against BICD2. Scale bars are 500 μm. b. Coronal cryo-sections of E17.5 cortices from Nex-KO, Emx1-KO and Nex-WT/Emx1-WT mice were stained against BICD2 (red). DAPI is shown in blue. Scale bars are 100 μm. c. Zooms from BICD2 staining shown in (b). d + e. Western Blots (WBs) of whole E17.5 cortex lysates from Nex-KO, Emx1-KO, and their control littermates Nex-WT and Emx1-WT. WBs were stained with commercial anti-BICD2 (atlas), home-made anti- BICD2 (#2294), home-made anti-BICD1/2(#2293), anti-actin or anti-tubulin. M = marker. Molecular weight of the marker bands are indicated on the left in kDa. (N = 3). f. Radial diameter of the cortex from ventricular to pial surface of Nex-WT, Nex-KO, Emx1-WT and Emx1-KO mice (in μm) (N = 16–17). *** p < 0.001, ns = not significant; error bars are ±SEM.CP: cortical plate, SVZ: subventricular zone, VZ: ventricular zone. Used test: Kruskal Wallis test with Dunn’s multiple comparisons. [file 40478_2019_827_MOESM1_ESM.pdf]

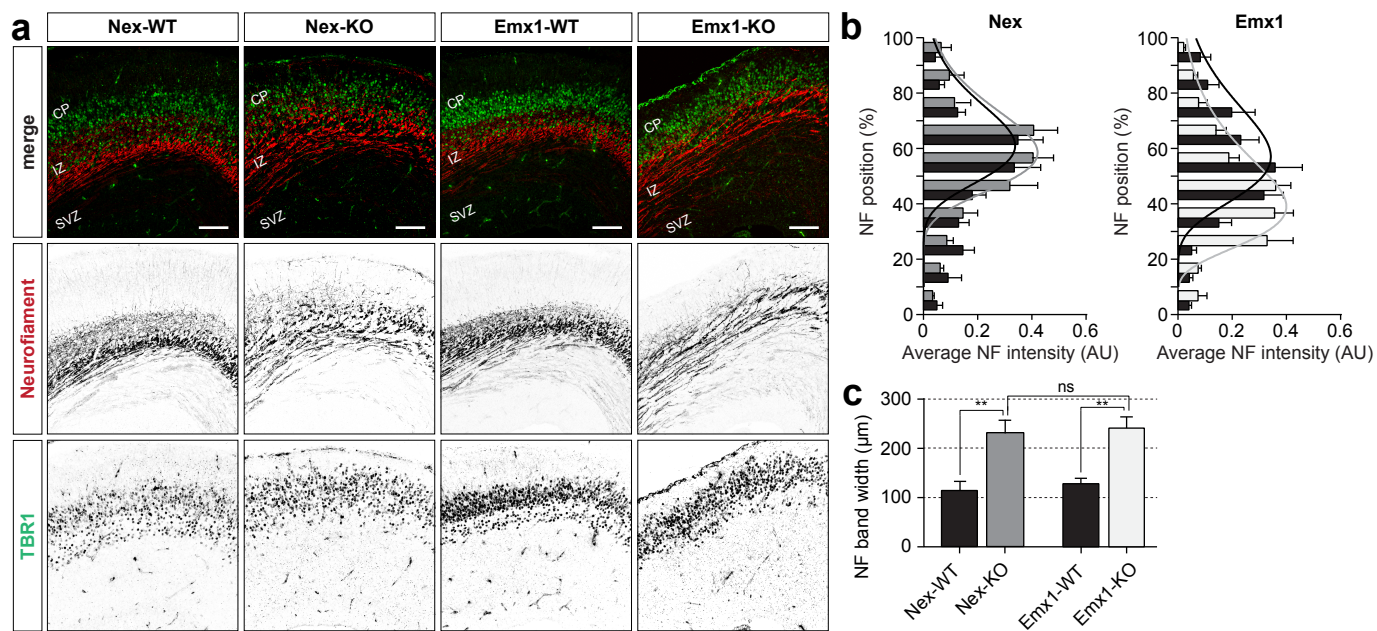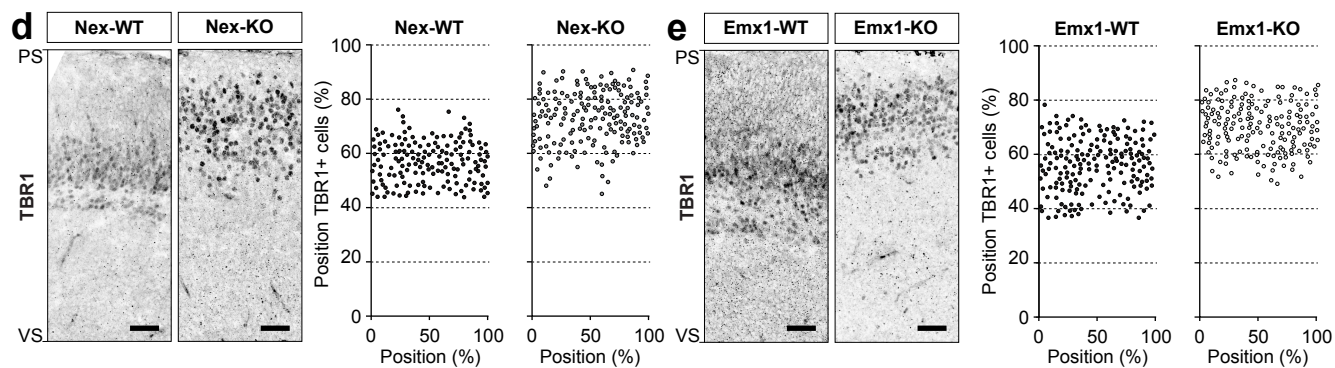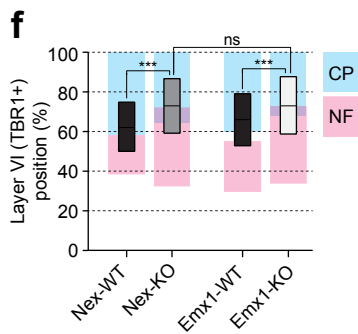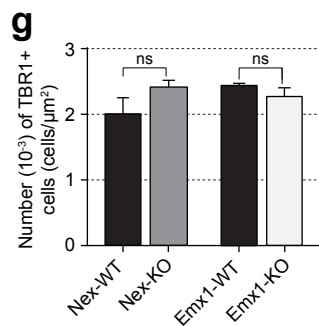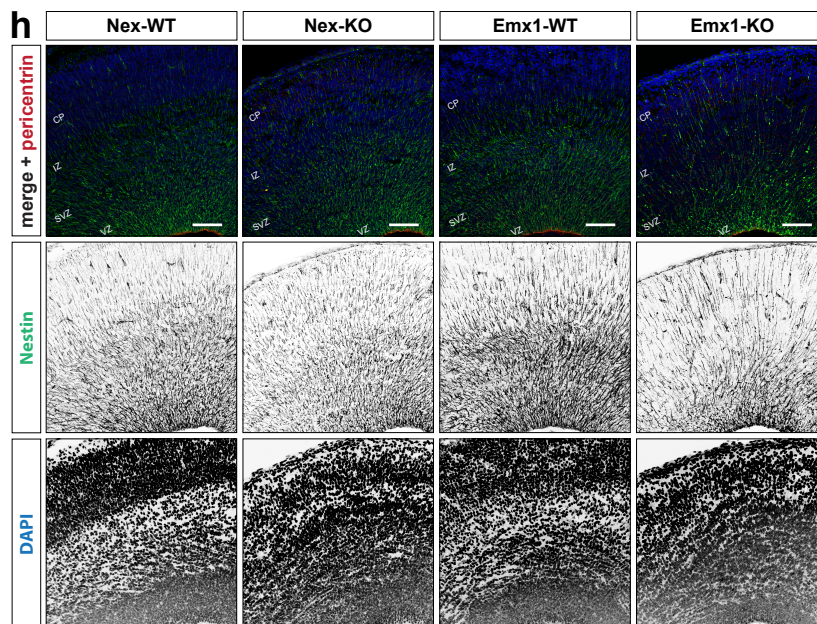

Supplement: Supplementary file 2 — Additional file 2: Fig. S2. Cell-type specific depletion of BICD2 does not affect migration of first-born CP neurons but impairs the axonal organization of contra-lateral and cortico-fugal projecting neurons in the IZ. a. Coronal cryo-sections of E17.5 cortices from cell-type-specific conditional Bicd2 KO mice and their control littermates - Bicd2fl/fl;Nex-Cre+/− (=Nex-KO), Bicd2fl/fl;Emx1-Cre+/− (=Emx1-KO), Bicd2fl/fl;Nex-Cre−/− (=Nex-WT) and Bicd2fl/fl;Emx1-Cre−/− (=Emx1-WT) respectively - were stained against Neurofilament heavy chain (NF) (red) and the cortical layer VI marker TBR1 (green). Scale bars are 100 μm. b. Quantification of the relative frequency of NF+ axons over the cortical longitude from ventricular (VS) to pial surface (PS) (%, binned in centers) and their gaussian distribution and the average NF intensity (AU) for Nex-WT and Nex-KO mice (left) and Emx1-WT and Emx1-KO mice (right). c. Width of the band with NF+ axons in μm (N = 4). d + e. Selected area from VS to PS and 156.3 μm width (left panels) and graphical representation of the relative position of TBR1+ cells over the cortical longitude from VS to PS (in %) and 156.3 μm in width (in %) (right panels) for Nex-WT and Nex-KO mice (d) and Emx1-WT and Emx1-KO mice (e). Scale bars in left panels are 50 μm. f. Relative position of TBR1+ layer VI neurons in the CP and NF+ axons in the IZ over the cortical longitude from VS to PS (in %). Bar represents average top, middle and bottom of the TBR1+ band (N = 3, n = 122–204). g. Number (10− 3) of TBR1+ cells per μm2 (N = 3, n = 122–204). h. Coronal cryo-sections of E17.5 cortices from Nex-KO, Emx1-KO, Nex-WT and Emx1-WT were stained against Nestin (green) to mark the fibers in RGP processes and Pericentrin (red) to mark the centrosomes. Scale bars are 100 μm. CP: cortical plate, IZ: intermediate zone, PS: pial surface, SVZ: subventricular zone, VS: ventricular surface, VZ: ventricular zone. *** p < 0.001, ** p < 0.005, ns = not significant; error bars a [file 40478_2019_827_MOESM2_ESM.pdf]

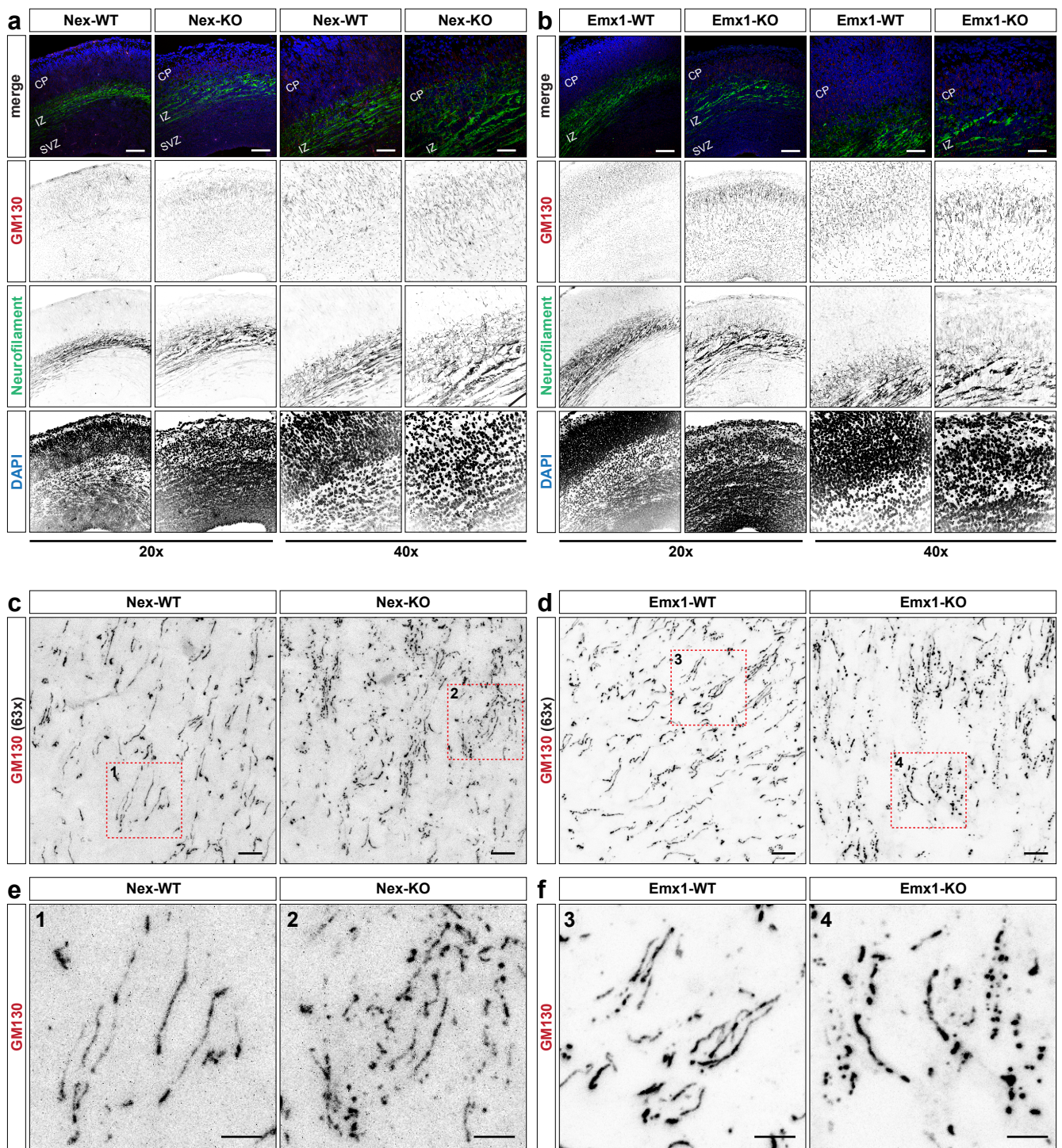

Supplement: Supplementary file 3 — Additional file 3: Fig. S3. BICD2 is essential for proper Golgi organization in CP neurons. a + b. Coronal cryo-sections of E17.5 cortices from Bicd2fl/fl;Nex-Cre−/− (=Nex-WT) and Bicd2fl/fl;Nex-Cre+/− (=Nex-KO) mice (a) and Bicd2fl/fl;Emx1-Cre−/− (=Emx1-WT) and Bicd2fl/fl;Emx1-Cre+/− (=Emx1-KO) mice (b) were stained against the trans-Golgi marker GM130 (red) and Neurofilament heavy chain (green) to indicate the IZ. DAPI is shown in blue. For each genotype confocal images in 20x (left) and 40x (right) are shown; scale bars are 100 μm and 50 μm, respectively. c + d. 63x zoom confocal images with GM130 stained trans-Golgi in the CP. Scale bars are 10 μm (c) and 50 μm (d). CP: cortical plate, IZ: intermediate zone, SVZ: subventricular zone. [file 40478_2019_827_MOESM3_ESM.pdf]

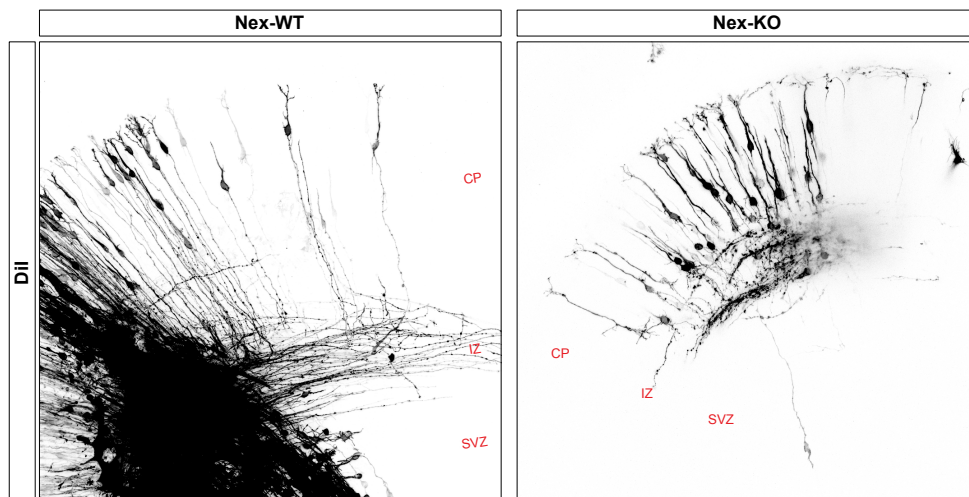

Supplement: Supplementary file 4 — Additional file 4: Fig. S4. DiI labeling of single neurons. Lightly fixed coronal brain sections from E17.5 Nex-WT and Nex-KO mice. DiI crystals were placed in the IZ to label and visualize individual neurons. CP: cortical plate, IZ: intermediate zone, SVZ: subventricular zone. [file 40478_2019_827_MOESM4_ESM.pdf]

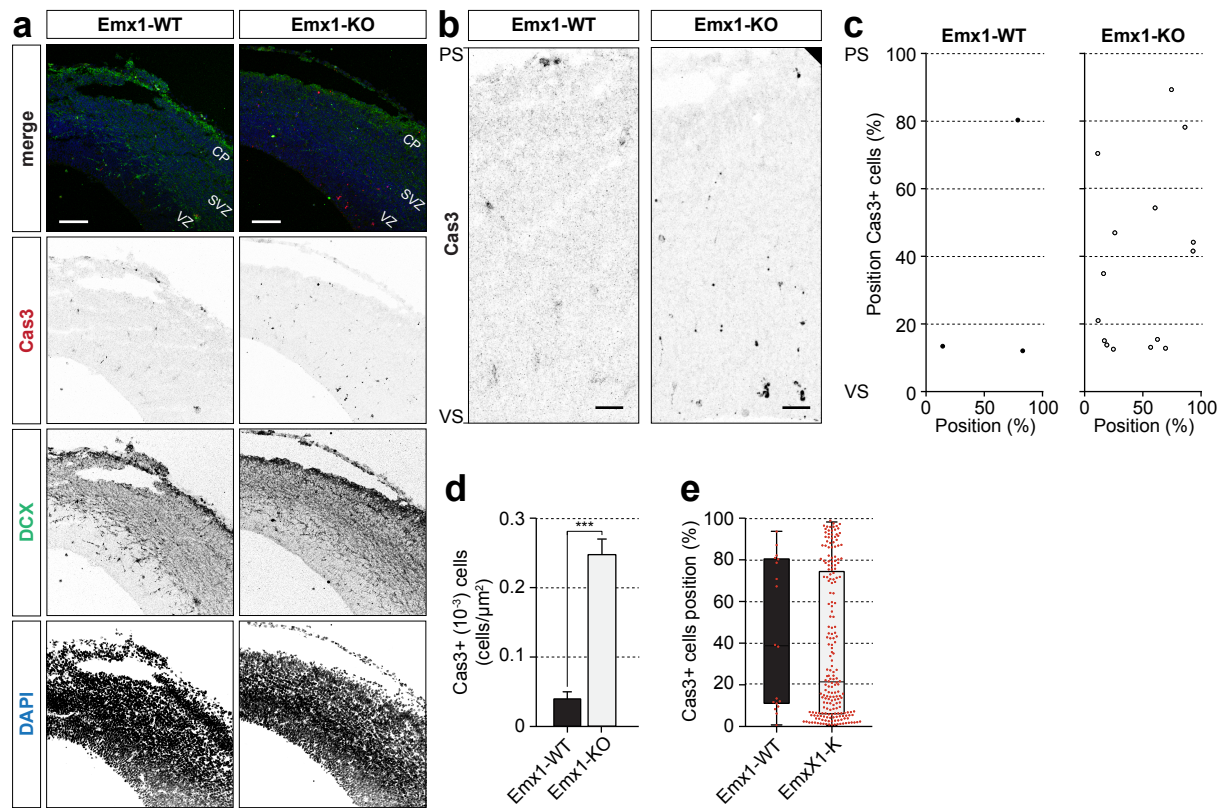

Supplement: Supplementary file 5 — Additional file 5: Fig. S5. Depletion of BICD2 in cortical cells results in an increase of apoptotic cell death in progenitor cell layers at E14.5. a. Coronal cryo-sections of E14.5 cortices from cell-type-specific conditional Bicd2 KO mice Emx1-Cre+/− (=Emx1-KO) and their control littermates Bicd2fl/fl;Emx1-Cre−/− were stained against apoptotic marker Caspase-3 (Cas3) (red) and Doublecortin (DCX) as early neuronal marker (green). DAPI is shown in blue. Scale bars are 100 μm. b. Zoom of Caspase-3 staining shown in (a). Scale bars are 50 μm. c. Graphical representation of the relative position of Cas3+ cells over the cortical longitude from ventricular (VS) to pial surface (PS) (both in %). d. Number (10− 3) of Cas3+ cells per μm2 (N = 6–12, n = 0–21). e. Cas3+ cell distribution as relative position over the cortical longitude from VS to PS (in %) for Emx1-WT and Emx1-KO mice. Red circles are individual Cas3+ cell locations of representative samples (N = 6–12, n = 0–21). *** p < 0.001; error bars are ±SEM. used test: unpaired t-test (d), Mann Whitney U test (e). [file 40478_2019_827_MOESM5_ESM.pdf]
