# Supplementary material for: Dynein activating adaptor BICD2 controls radial migration of upper-layer cortical neurons in vivo
Source: Acta Neuropathol Commun. 2019 Oct 26;7:162. doi: 10.1186/s40478-019-0827-y (PMC6815425; doi:10.1186/s40478-019-0827-y)
Supplement: Supplementary file 6 — Additional file 6: Table S1. Cloning and sequencing primers. [file 40478_2019_827_MOESM6_ESM.docx]

**Table S1**

| Bicd2S107L_fw | gcctgatccaggagttggcctccaaggag |
| --- | --- |
| Bicd2S107L_rev | gctccttggaggccaactcctggatcaggc |
| Bicd2E774G_fw | ggcagctgccgaggacgggaagaagacccttaactctctg |
| Bicd2E774G_rev | cagagagttaagggtcttcttcccgtcctcggcagctgcc |
| Bicd2R694C_fw | ccgccgggaacctatgaacatc |
| Bicd2R694C_rev | ccagtcgacctacaggctcggtgaggctggcttggc |
| Bicd2fw_P1 | gctgccgctgcatctcatccag |
| Bicd2rev_P6 | ccgccgggaacctatgaacatc |
| Bicd2_seq1 | gtgggaggttttttaaagcaagt |
| Bicd2_seq2 | gctgccgctgcatctcatccag |
| Bicd2_seq3 | ccgccgggaacctatgaacatc |
